# Structural changes in perineuronal nets and their perforating GABAergic synapses precede motor coordination recovery post stroke

**DOI:** 10.1186/s12929-023-00971-x

**Published:** 2023-09-01

**Authors:** Egor Dzyubenko, Katrin I. Willig, Dongpei Yin, Maryam Sardari, Erdin Tokmak, Patrick Labus, Ben Schmermund, Dirk M. Hermann

**Affiliations:** 1 Department of Neurology and Center for Translational Neuro- and Behavioral Sciences (C-TNBS), University Hospital Essen, Hufelandstraße 55, 45122 Essen, Germany; 2 Group of Optical Nanoscopy in Neuroscience, Max Planck Institute for Multidisciplinary Sciences, City Campus, Hermann-Rein-Str. 3, 37075 Göttingen, Germany

**Keywords:** Extracellular matrix, Stroke recovery, Synaptic rewiring, Parvalbumin interneurons, Fluorescence nanoscopy, Neuroinflammation

## Abstract

**Background:**

Stroke remains one of the leading causes of long-term disability worldwide, and the development of effective restorative therapies is hindered by an incomplete understanding of intrinsic brain recovery mechanisms. Growing evidence indicates that the brain extracellular matrix (ECM) has major implications for neuroplasticity. Here we explored how perineuronal nets (PNNs), the facet-like ECM layers surrounding fast-spiking interneurons, contribute to neurological recovery after focal cerebral ischemia in mice with and without induced stroke tolerance.

**Methods:**

We investigated the structural remodeling of PNNs after stroke using 3D superresolution stimulated emission depletion (STED) and structured illumination (SR-SIM) microscopy. Superresolution imaging allowed for the precise reconstruction of PNN morphology using graphs, which are mathematical constructs designed for topological analysis. Focal cerebral ischemia was induced by transient occlusion of the middle cerebral artery (tMCAO). PNN-associated synapses and contacts with microglia/macrophages were quantified using high-resolution confocal microscopy.

**Results:**

PNNs undergo transient structural changes after stroke allowing for the dynamic reorganization of GABAergic input to motor cortical L5 interneurons. The coherent remodeling of PNNs and their perforating inhibitory synapses precedes the recovery of motor coordination after stroke and depends on the severity of the ischemic injury. Morphological alterations in PNNs correlate with the increased surface of contact between activated microglia/macrophages and PNN-coated neurons.

**Conclusions:**

Our data indicate a novel mechanism of post stroke neuroplasticity involving the tripartite interaction between PNNs, synapses, and microglia/macrophages. We propose that prolonging PNN loosening during the post-acute period can extend the opening neuroplasticity window into the chronic stroke phase.

**Graphical Abstract:**

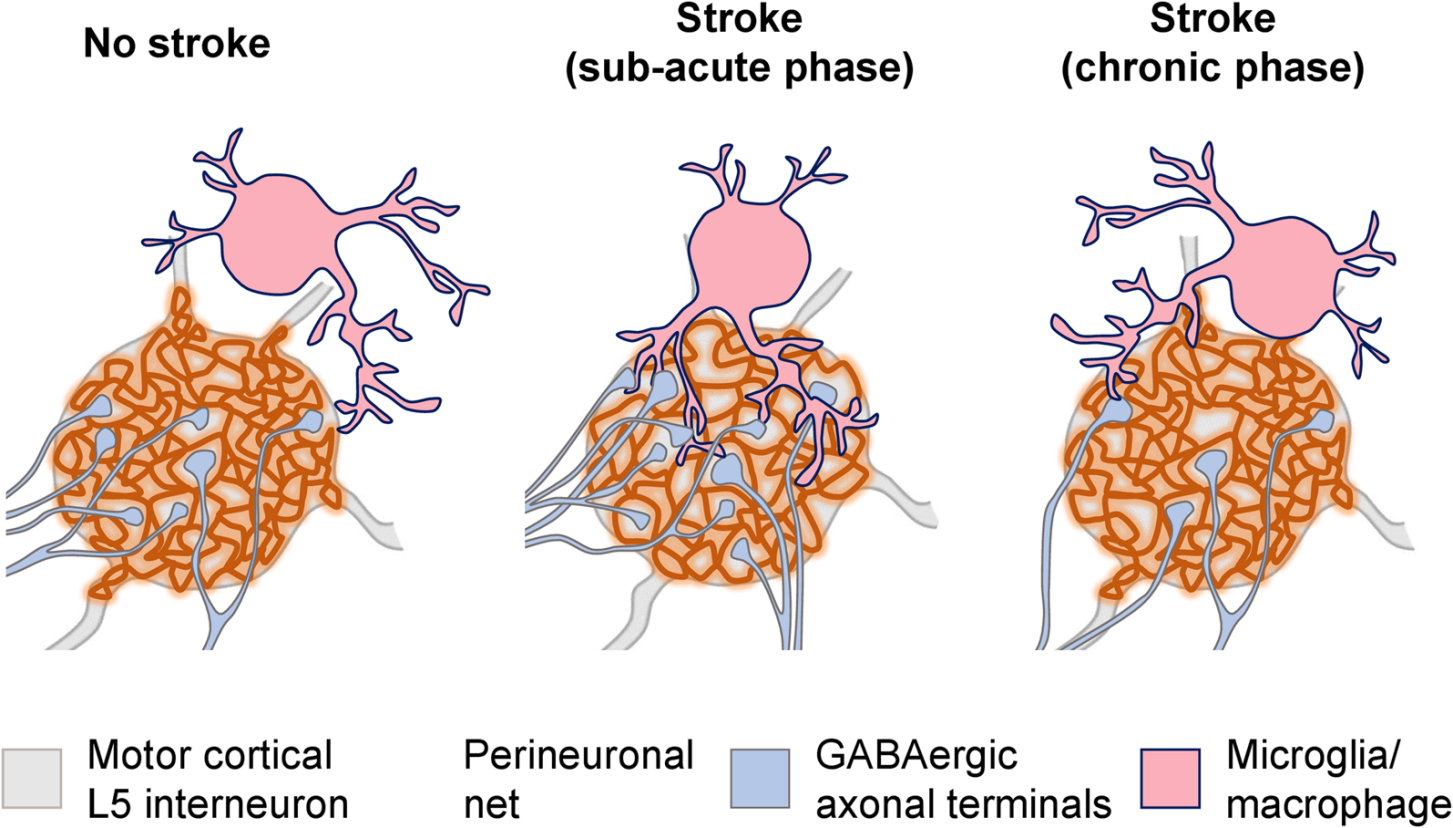

**Supplementary Information:**

The online version contains supplementary material available at 10.1186/s12929-023-00971-x.

## Highlights

- PNNs are degraded partially and transiently post stroke.
- Transient attenuation of PNNs correlates with GABAergic synapse remodeling.
- Transient attenuation of PNNs precedes functional recovery post stroke.
- Activated microglia/macrophages preferentially contact PNN-coated neurons post stroke.

**Supplementary Information:**

The online version contains supplementary material available at 10.1186/s12929-023-00971-x.

## Background

Brain remodeling is essential for regaining compromised motor activity and coordination post stroke. Neurological recovery after stroke involves several neuroplasticity mechanisms including corticospinal tract rewiring [37], sprouting of interhemispheric cortico-cortical projections [42], and remodeling of local intracortical connectivity [7]. Motor cortical activity defining motor commands during skilled limb movements is selectively distributed across neurons with distinct projection patterns [50], suggesting that long-range and local connectivity reorganization is similarly pivotal for a successful recovery after stroke. Experimental treatments promoting pyramidal tract plasticity have been proposed post stroke [53], but stimulating the plasticity of intracortical projections requires a deeper understanding of local connectivity changes in the motor cortex. Cortical oscillations underlying motor learning arise from the fast-spiking activity of layer 5 (L5) interneurons [48], which are the main source of inhibition in neocortical microcircuits [46, 49]. Although motor cortical L5 interneurons are critical for controlling coordinated movements, their involvement in post stroke brain remodeling has not been systematically studied to the best of our knowledge.

Cortical L5 fast-spiking interneurons express parvalbumin (PV), potassium channels with rapid activation and deactivation kinetics (Kv3.1) and are coated with perineuronal nets (PNNs) on the extracellular side [11, 35, 45]. PNNs are condensed lattice-like layers of extracellular matrix (ECM) composed of multiple macromolecules including hyaluronic acid, chondroitin sulfate proteoglycans (CSPGs), and link proteins [10, 61]. These polymeric assemblies propagate to the extracellular space (ECS) and are anchored to the neuronal surface via hyaluronic acid synthases [30, 41]. PNNs are rigid structures resistant to chemical decomposition [17] that are formed in an activity-dependent manner [18] and restrict synaptic plasticity [29, 52]. PNNs compartmentalize neuronal surface, limit astrocyte-neuron direct membrane contacts and new synapse formation, stabilize existing connectivity, and hypothetically contribute to potassium buffering (for review see [20, 27]). Thereby, PNNs maintain the fast-spiking properties of interneurons [63] and inhibitory control in neuronal networks [19].

In the adult brain, CSPGs within PNNs restrict neuronal plasticity by inhibiting axonal sprouting [31], and experimental approaches involving non-specific ECM digestion have been shown to promote functional recovery post stroke [32]. It is unlikely though that the complete removal of ECM and PNNs in particular can be implemented clinically because it induces epileptiform activity [2, 3] and impairs memory formation [33, 39]. Therefore, understanding the impact of more delicate PNN alterations on neuroplasticity is imperative for developing novel therapies targeting brain ECM. In a previous study, we developed a method allowing for the topological quantification of PNN morphology based on superresolution fluorescence microscopy [21]. We showed that despite their rigidity, PNNs are subject to subtle modulation post stroke and anticipated that their transient loosening can support brain remodeling. In this work, we have further elaborated our method and investigated PNN morphology with nanoscale resolution and linked it to synaptic remodeling and neurological recovery post stroke. Moreover, we explored the impact of coherent rearrangements in PNNs and their perforating synapses after ischemia in mice with induced stroke tolerance, providing a comprehensive understanding of ECM-mediated neuroplasticity following strokes of varying severity.

## Materials and methods

### Legal issues, animal housing, and randomization

Experimental procedures were conducted in accordance with European Union (Directive 2010/63/EU) guidelines for the care and use of laboratory animals and approved by the local government (Bezirksregierung Düsseldorf). C57BL/6j mice were kept in groups of 5 animals per cage, inverse 12/12 h light/dark cycle, and access to food and water ad libitum. All efforts were made to reduce the number of animals in the experiments. The groups were randomly assigned using dummy names, and the experimenters were blinded to group coding during sample preparation, data acquisition, and analysis.

### Cerebral ischemia, ischemic tolerance induction, and tissue collection

Wildtype male C57/Bl6 mice at the age of 2 months were randomly assigned into three groups: ischemic stroke, preconditioning and ischemic stroke, and naive control. Each group included 7 animals. Focal cerebral ischemia was induced by transient left-sided intraluminal transient middle cerebral artery occlusion (tMCAO) for 30 min as described previously [22]. In brief, mice were anesthetized with 1.5% v/v isoflurane (carrier gas was N_2_O with 30% v/v O_2_) and 150 µl of 15 µg/ml buprenorphine was injected subcutaneously. After exposing and ligating the lower part of the left common carotid artery (CCA), a silicon-coated nylon monofilament was introduced through a fine incision and advanced until the bifurcation of the middle cerebral artery (MCA). The cessation of blood supply in the MCA territory was verified by measuring laser Doppler flow (LDF). After 30 min, the filament was removed to induce reperfusion, which was controlled by LDF recording. The tMCAO procedure resulted in highly reproducible ischemic lesions located in the striatum and adjacent cortical areas, but not the motor cortex, which allowed for the analysis of PNN morphology and synaptic remodeling in this crucial brain region. Importantly, the produced infarcts had similar volumes as in our previous studies [21, 57], and ischemic animals showed all characteristic aspects of stroke lesion, including reactive gliosis, behavioral deficits, and post-acute brain atrophy.

**Fig. 1:**
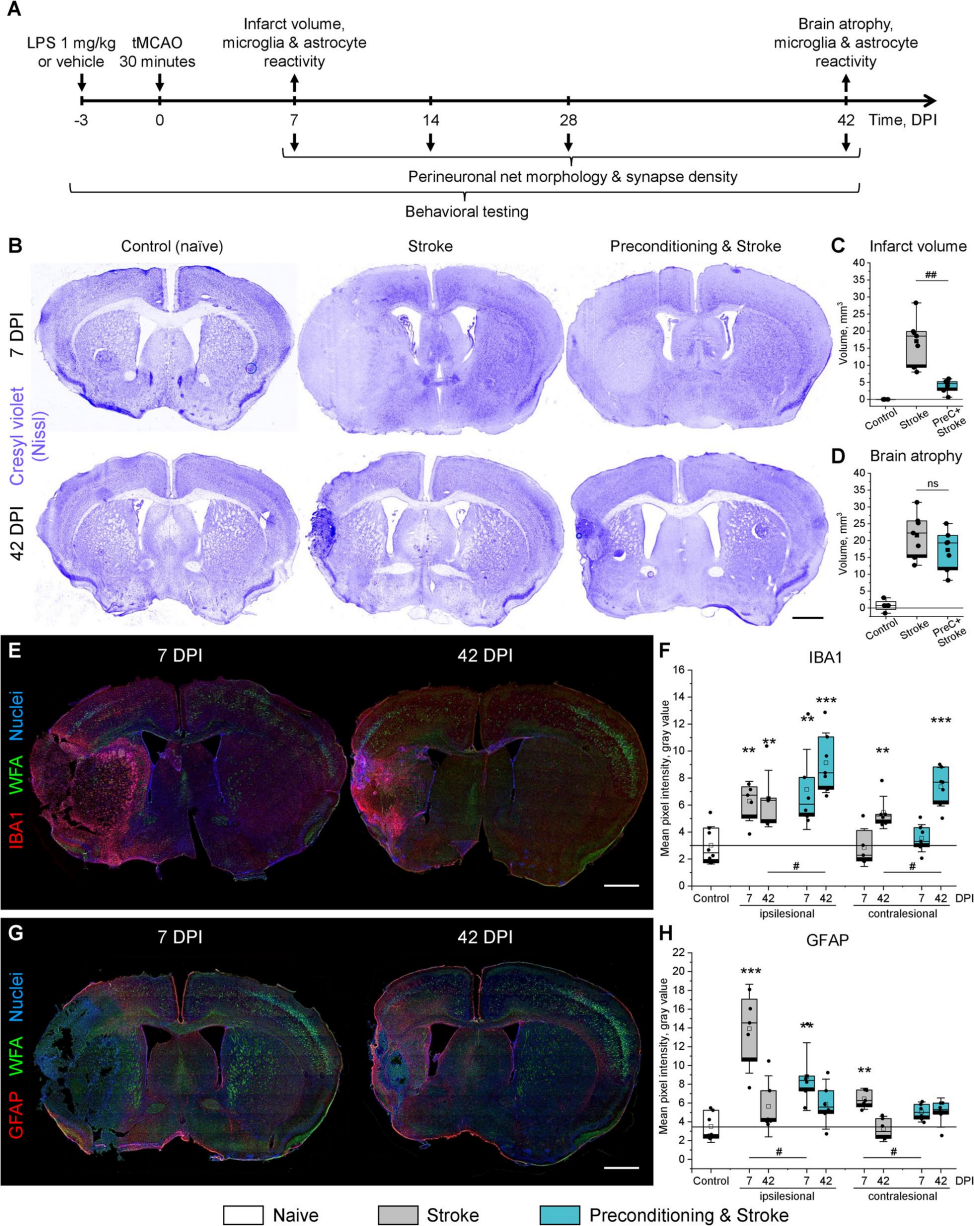
Brain damage and reactive gliosis induced by focal cerebral ischemia. **A** Timeline and experimental endpoints. **B** Cresyl violet (Nissl) staining shows focal infarcts at 7 DPI, quantified in **C**, and brain atrophy at 42 DPI, quantified in **D**. **E** Representative WFA (green) and IBA1 (red) staining at 7 and 42 DPI. **F** IBA1 immunoreactivity in the motor cortex L5. **G** Representative WFA (green) and GFAP (red) staining at 7 and 42 DPI. **H** GFAP immunoreactivity in the motor cortex L5. **E**, **G** White squares outline 600 × 600 µm regions of interest selected for analysis. Nuclei (blue) were stained with DAPI. **C**, **D**, **F**, **H** Graphs are box plots with data as dots, means as squares, medians as lines, interquartile ranges as boxes, and whiskers showing SD. Asterisks and hashes denote significant differences with the control and stroke groups, correspondingly, as indicated by two-way ANOVA and t-tests (*^,#^p < 0.05, **^,##^p < 0.01, ***p < 0.001), n = 7. Scale bars, 1 mm. DPI, days post ischemia; ns, not significant

Ischemic tolerance was induced by inflammatory preconditioning with 1 mg/kg LPS that was injected intraperitoneally 3 days before tMCAO to trigger a robust peripheral immune response that we reported previously [57]. Experimental endpoints to evaluate post stroke brain remodeling during post-acute and chronic stroke phases were 7, 14, 28, and 42 days post ischemia (DPI) (Fig. 1A). Upon reaching the pre-defined endpoints, animals were anesthetized with 100 µl of ketamine-xylazine (3:1) and sacrificed by transcardiac perfusion with 4% paraformaldehyde (PFA) in normal saline. The brains were removed and immersed in 4% PFA solution for 12 h at 4 °C. Tissues were cryoprotected in sucrose gradient solutions (10–30%), carefully dried, frozen, and stored at − 80 °C until further processing.

### Infarct volume and brain atrophy measurement

Coronal sections of the brain (20 µm thick) were collected at 500 µm intervals across the forebrain using a Leica CM1950 cryostat and placed onto cold microscope slides (ThermoFisher Scientific, Cat# J1800AMNT). The sections were stained with Cresyl violet (that is, Nissl) and scanned using the AxioObserver Z1 microscope (objective Plan-Apochromat 10 × /0.45 M25; Zeiss, Jena, Germany). The infarct volume (IV) was measured at 7 DPI as IV = Σ(IA*Δ), where IA is the infarcted area on a section and Δ is the distance between sections. Brain atrophy at 42 DPI was determined by subtracting the areas of surviving tissue in the ipsilesional hemisphere from the areas of the contralesional hemisphere. Atrophy volume (AV) was calculated as AV = Σ(AA*Δ), where AA is the atrophied area on a section and Δ is the distance between sections.

### Neurological deficits and motor performance tests

General and focal neurological deficits were analyzed using Clark’s neuroscore [12] daily until 7 DPI, every 3 days until 14 DPI, and weekly until 42 DPI. Post stroke recovery of motor activity and coordination was assessed by tight rope tests at baseline, 7, 14, 21, 28, 35, and 42 DPI as described previously [57]. In brief, the tight rope test measures the time until the animals reach the platform from the middle of a 60-cm-long rope. Mice were pre-trained for 3 days before tMCAO ensuring that they were able to reach the platform within ten seconds.

### Immunohistochemical procedures

Free-floating coronal Sects. (30 μm thick) were obtained at the level of bregma + 0.5 to + 1 mm using a Leica CM1950 cryostat and stored until use at − 20 °C in 1:1 mixture of phosphate buffer saline (PBS) and ethylene glycol with 1% polyvinyl pyrrolidone. For immunohistochemistry, the sections were rinsed with 0.1 M PBS and permeabilized with 0.3% w/v Triton X-100 in PBS. Non-specific antibody binding was blocked by applying a mixture of 10% v/v ChemiBLOCKER (Cat# 2170, Millipore, Burlington, MA, U.S.A.) and 5% v/v normal donkey serum in PBS for 12 h at room temperature with gentle agitation. Sections were incubated with primary antibodies for 48 h at 4 °C in PBS with 0.01% w/v Triton X-100. Astrocytes and microglia/macrophage cells were labeled using rat anti-GFAP (1:300; Cat# 13-0300, Thermo Fisher Scientific, Waltham, MA, U.S.A) and rabbit anti-IBA1 (1:500; Cat# 019-19741, Wako, Neuss, Germany) or guinea pig anti-IBA1 (1:300, Cat# 234308, Synaptic Systems, Göttingen, Germany) antibodies. Interneurons were detected with rabbit anti-parvalbumin (1:500; Cat# 195002, Synaptic Systems) and rabbit anti-Kv3.1b (1:1000, Cat# APC-014, Alomone Labs, Jerusalem, Israel). PNNs were labeled with biotinylated *Wisteria floribunda* agglutinin (WFA) (1:100, B-1355, Vector Laboratories, Burlingame, USA). Glutamatergic and GABAergic synaptic terminals were detected using guinea pig anti-VGLUT1 (1:500, Cat# 135304, Synaptic Systems) and guinea pig anti-VGAT (1:500, Cat# 131103, Synaptic Systems) antibodies. For fluorescence detection, we used secondary antibodies conjugated to Alexa 488, 594, and 647 or streptavidin conjugated to Atto 495, Atto 590, or Abberior STAR RED dyes. Nuclei were counterstained with DAPI (1:1000, D1306, ThermoFisher). The bound antibodies were stabilized by incubating the sections in 2% w/v PFA for 30 min at room temperature. For high-resolution imaging with confocal, SR-SIM, and STED microscopy with oil immersion objectives, the refractive index of stained tissues was adjusted to 1.5 using 2,2’-thiodiethanol (TDE, Cat# 166782, Merck, Darmstadt, Germany), which is widely used for tissue clearing [13].

### Quantification of glial and neuronal markers

The expression of GFAP and IBA1 markers was analyzed using an AxioObserver Z1 microscope (objective Plan-Apochromat 10 × /0.45 M25; Zeiss). In the whole-section images obtained by tiling, 600 × 600 µm regions of interest (ROIs) were selected in the motor cortical layer 5 as shown in Fig. 1E, G, and the mean pixel intensity was measured using ImageJ (National Institutes of Health, Bethesda, MD, U.S.A.). In each animal, four images obtained from two adjacent brain sections were analyzed in the ipsilesional and contralesional motor cortex.

The cell density of interneurons expressing PV, Kv3.1, and PNNs was quantified manually in 600 × 600 × 10 µm ROIs obtained in the motor cortical L5 regions using the LSM 710 confocal microscope (Zeiss, 20 × Plan Apochromat objective, NA 0.8, pixel size 0.21 µm). In each animal, four image stacks obtained from two adjacent brain sections were analyzed in the ipsilesional and contralesional motor cortex.

### Quantification of synaptic inputs and surface contacts between neurons and microglia/macrophages

The number of axonal terminals perforating PNNs was quantified in 33 × 33 × 5 µm z-stacks obtained with high-resolution confocal microscopy using the LSM 710 microscope (Zeiss, 100 × alpha Plan-Apochromat objective, NA 1.46, voxel size 60 × 60 × 500 nm). ROIs were positioned in the motor cortex L5 (see Additional file 4: Fig. S1) containing a single PNN-coated neuron. Synaptic terminals expressing VGAT or VGLUT1 that associated with WFA labeling were counted using an automated ImageJ routine (see Additional file 1). In each animal, four ROIs obtained from two adjacent brain sections were analyzed.

The surface of microglia/macrophage-neuron contacts was quantified in 75 × 75 × 10 µm z-stacks obtained with high-resolution confocal microscopy using the LSM 710 microscope (Zeiss, 63 × alpha Plan-Apochromat objective, NA 1.4, voxel size 70 × 70 × 450 nm). ROIs were positioned in the motor cortex L5 containing a single PNN-coated neuron. Surfaces representing IBA1 (microglia/macrophages) and Kv3.1 (fast-spiking interneurons) labeled cells were generated by automated thresholding with IMARIS 9.9 software (Oxford Instruments, Stockholm, Sweden) using the standard surfaces function. The area of contact between cells was quantified as the intersection between IBA1 and Kv3.1 surfaces.

### Microscopy techniques and resolution measurements

The morphology of WFA-labeled PNNs was analyzed using the four 3D imaging methods: two-photon excitation (2P), confocal, superresolution structured illumination (SR-SIM), and 3D stimulated emission depletion (STED) microscopy.

2P microscopy of PNNs labeled with Atto 495 was performed using a Leica TCS SP8 (25 × HCX IRAPO L 25 × water immersion objective, NA 0.95) microscope. ROIs (92 × 92 × 11.5 µm, voxel size 90 × 90 × 480 nm) were scanned as z-stacks. The excitation laser (Titanium:Sapphire Chameleon Vision II) was tuned to 940 nm, and the output power was 1.712 W. Emitted fluorescence (500–570 nm detection wavelength) was collected using a hybrid detector. Imaging resolution was estimated by measuring the full width at half maximum (FWHM) of Ø 100 nm TetraSpeck microspheres (Cat# T7279, Thermo Fisher Scientific) using the same microscope settings as for PNN imaging.

Confocal microscopy of PNNs labeled with Atto 590 was performed using an LSM 710 (100 × alpha Plan-Apochromat objective, NA 1.46) microscope. ROIs (65.4 × 65.4 × 5.5 µm, voxel size 60 × 60 × 350 nm) were scanned as z-stacks. An excitation laser (561 nm DPSS) was used at 10% of maximum output power to reduce photobleaching. The confocal pinhole size (585–655 nm detection wavelength) was adjusted to 1 Airy unit. Imaging resolution was estimated by measuring FWHM of Ø 100 nm TetraSpeck microspheres (Cat# T7279, Thermo Fisher Scientific) using the same microscope settings as for PNN imaging.

SR-SIM microscopy of PNNs labeled with Atto 590 or Abberior Star RED was performed using a Carl Zeiss Elyra PS.1 (100 × alpha Plan-Apochromat objective, NA 1.46) microscope. ROIs (49.4 × 49.4 × 6.7 μm, voxel size 20 × 20 × 100 nm) were scanned as z-stacks. Excitation lasers (561 and 642 nm OPSL) were used at 5% of maximum output power to reduce photobleaching. We used the 51 µm grid (5 rotations, 5 phases) for 585–655 nm and 649–755 nm detection wavelengths. The output superresolution images were computed using the automatic processing mode using the Zen Black software (Zeiss). Imaging resolution was estimated by measuring FWHM of Ø 100 nm TetraSpeck microspheres (Cat# T7279, Thermo Fisher Scientific) using the same microscope settings as for PNN imaging.

STED microscopy of PNNs labeled with Abberior Star RED was performed using the custom-built setup [67] at the Max Planck Institute for Multidisciplinary Sciences in Göttingen, Germany. We used an oil immersion objective (HCX PL APO 100 ×/1.40 OIL STED, Leica Microsystems). ROIs (25 × 25 × 5 μm, voxel size 30 × 30 × 100 nm) were scanned as z-stacks. Pixel dwell time was 10 µs. Pulsed excitation light of 650 nm was spectrally filtered from a white light source [67] and applied with an average power of 2.7 µW at the back focal plane of the objective. The STED laser (Katana 08 HP, OneFive GmbH, Regensdorf, Swiss), providing nanosecond pulses at 775 nm, was employed with a power of 221 mW. The STED beam was shaped by a spatial light modulator (Abberior Instruments) with a 2π-vortex and π phase delay for x/y- and z-depletion, respectively. The x/y- lateral and z-axial resolution was independently adjusted and estimated by measuring the FWHM of Ø 40 nm TransFluoSpheres (Ex/Em 633/720 nm, Cat# T8870, Thermo Fisher Scientific) using the same microscope settings as for PNN imaging.

### Reconstruction and analysis of PNN morphology

Structural organization of PNNs was visualized by superresolution microscopy and their morphology was analyzed using quantitative graph-based computational reconstruction. The previously reported method [21] was optimized to improve imaging resolution and reliability of quantifications. The new optimized MATLAB code is provided here as Additional file 2. In each animal, a total of 8 individual PNNs were analyzed in the ipsilesional and contralesional motor cortex. In brief, the 3D image stacks obtained by superresolution STED or SR-SIM microscopy were imported into the IMARIS 9.9 program (Oxford Instruments) and local fluorescence intensity maxima were defined as net nodes representing PNN mesh vertices. The internode connections (edges) were reconstructed using the non-redundant nearest neighbor search algorithm in MATLAB. The resulting graphs were used to derive morphological metrics that characterize the structure of PNN facets. More specifically, we quantified average internode distances (*L)* indicating PNN facet size and the average node degrees (*D*) reflecting facet density.

### Statistical planning and analysis

Sample sizes were determined by power analysis (Additional file 3) in accordance with Institutional Animal Care and Use Committee (IACUC) guidelines [40] using the anticipated mean values, standard deviations, and effect sizes based on our previous studies [21, 55, 56]. To achieve 80% power (α = 0.05, β = 0.2), we enrolled 7 animals per group.

All quantitative data were presented as box plots indicating the mean (empty square), the median (line), 25–75% range (borders) and SD (whiskers) of data distribution. For all datasets, normal distribution was analyzed using Lilliefors, Kolmogorov–Smirnov, and Shapiro–Wilk tests. The differences between groups were evaluated using one-way (infarct volume and brain atrophy) or two-way (all other readouts) analysis of variance (ANOVA) and post hoc pairwise t-tests. For multiple comparisons, Bonferroni corrections were applied.

## Results

### Inflammatory preconditioning reduces the infarct size but not delayed brain atrophy

We investigated post stroke brain remodeling in mice with and without stroke tolerance induced by inflammatory preconditioning. Focal cerebral ischemia was induced by transient left-sided intraluminal occlusion of the middle cerebral artery (tMCAO) for 30 min resulting in highly reproducible ischemic lesions located in the striatum and adjacent cortical areas, but not the motor cortex. To induce ischemic tolerance, inflammatory preconditioning was performed by injecting 1 mg/kg LPS intraperitoneally 3 days before tMCAO (Fig. 1A), which triggered a robust peripheral immune response that we reported previously [57]. In agreement with previous studies [44, 54], the inflammatory preconditioning protected the brain tissue against ischemic injury and significantly reduced infarct volume at 7 days post ischemia (DPI), as indicated by Nissl staining quantifications (Fig. 1B, C). As a matter of fact, preconditioning with LPS did not mitigate the delayed brain atrophy at 42 DPI (Fig. 1B, D), suggesting a multifaceted nature of long-term brain remodeling that cannot be foreseen based solely on the acute lesion size.

### Inflammatory preconditioning alters glial responses post stroke

Post stroke reactive gliosis was evaluated in 600 × 600 µm ROIs located within motor cortex (Additional file 4: Fig S1) using IBA1 and GFAP immunohistochemistry detecting microglia/macrophages and reactive astrocytes, correspondingly (Fig. 1E, G). Focal cerebral ischemia triggered reactive gliosis activation not only in lesion-associated areas, but also in the contralesional motor cortex (Fig. 1E–H). Microglia/macrophage activation persisted at 42 DPI and was also observed in the contralesional hemisphere. Inflammatory preconditioning increased IBA1 immunoreactivity at 42 DPI, but not 7 DPI. In contrast, astrocytic reactivity at 7 DPI was reduced in the group exposed to both preconditioning and stroke, as indicated by GFAP immunoreactivity (Fig. 1H).

### Interneurons lose PNNs partially and transiently after stroke

Cortical L5 fast spiking interneurons are critical for oscillatory activity in the motor cortex, pyramidal tract output activity regulation, and motor control [23]. These cells express parvalbumin, potassium channels Kv3.1, and are coated with a pattern of aggregated extracellular matrix forming PNNs [35, 59]. PNNs regulate several types of neuroplasticity [8, 52, 58, 63], but their role in post stroke brain remodeling remains under investigated.

**Fig. 2:**
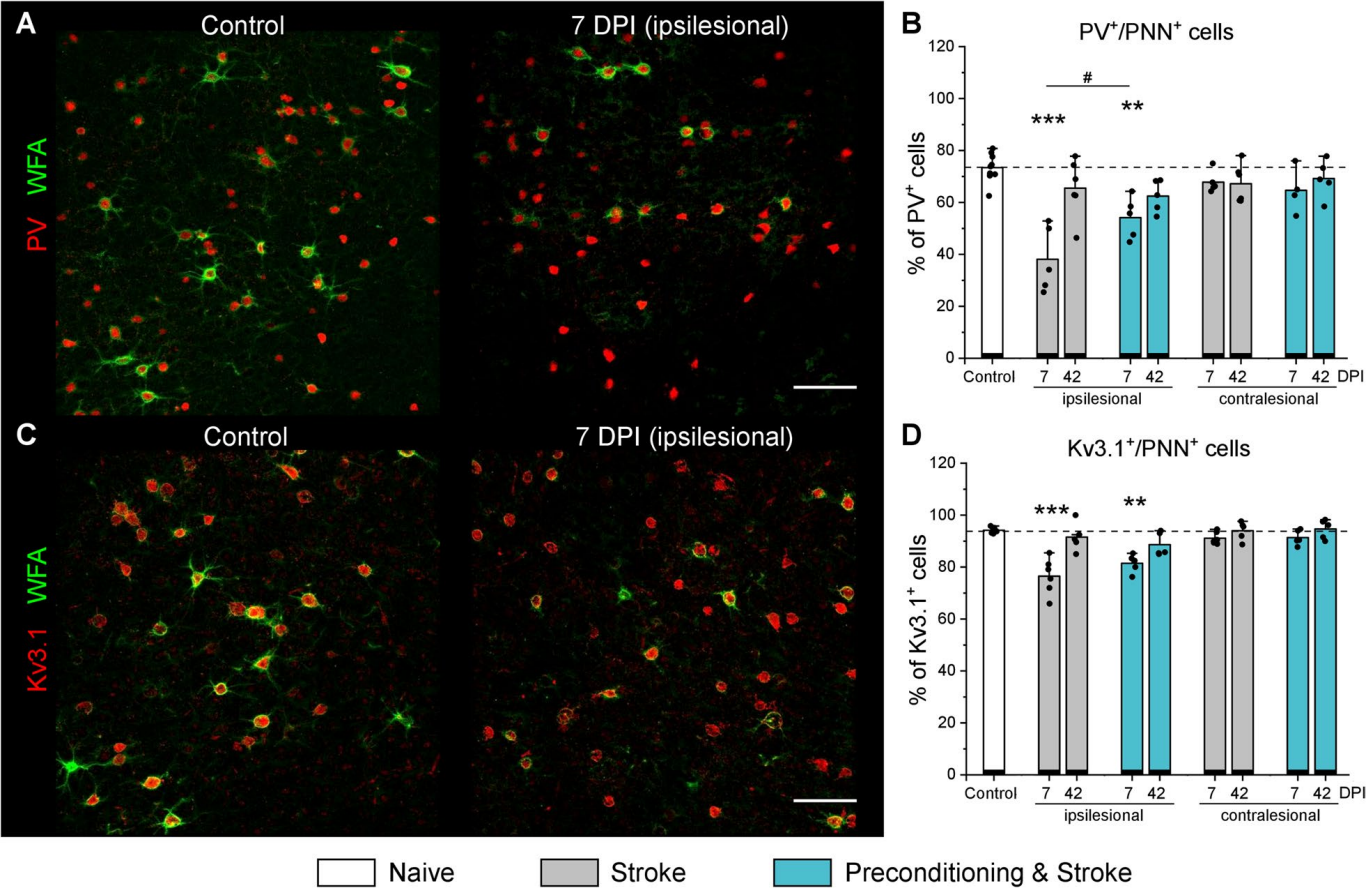
PNN expression in the motor cortex L5 post stroke. Representative immunolabeling of WFA (green) and (**A**) PV (red) or (**C**) Kv3.1 (red) shows PNN expression around motor cortical L5 interneurons. **B**, **D** Percentage of PV (**B**) and Kv3.1 (**D**) expressing neurons coated with PNNs. Graphs are bar plots showing mean ± SD and data as dots. Asterisks and hashes denote significant differences with the control and stroke groups, correspondingly, as indicated by two-way ANOVA and t-tests (^#^p < 0.05, **p < 0.01, ***p < 0.001), n = 7. Scale bars, 100 µm. DPI, days post ischemia; PV, parvalbumin

We analyzed the expression of PNNs around motor cortical L5 interneurons (ROIs are shown in Additional file 4: Fig S1) using the WFA agglutinin that binds glycan chains of extracellular proteoglycans enriched in PNNs [17, 34]. Co-labelling of parvalbumin (PV) and Kv3.1 with WFA (Fig. 2A, C) indicated that stroke reduced the expression of PNNs in the ipsilesional motor cortex L5 at 7 DPI (Additional file 4: Fig S2A). In healthy brains, 73.4 ± 5.5% PV^+^ and 93.5 ± 2.1% Kv3.1^+^ expressed PNNs, as evidenced by PV^+^/PNN^+^ (Fig. 2B) and Kv3.1^+^/PNN^+^ (Fig. 2D) cell quantifications. In mice exposed to stroke only, on average 34.3% PV^+^ and 15.3% Kv3.1^+^ neurons in the ipsilesional motor cortex lost PNN coatings during the first week post stroke. PNN expression was restored by 42 DPI. Inflammatory preconditioning reduced the transient loss of PNNs around PV^+^ neurons at 7 DP, compared with the stroke-only group (Fig. 2B). At all time points and in all conditions, the majority of PNN^+^ cells were also PV^+^ (96%) and Kv3.1^+^ (97%), and stroke altered neither PV^+^ nor Kv3.1^+^ cell density, as shown in Additional file 4: Fig S2.

### PNN morphology analysis requires superresolution imaging

**Fig. 3:**
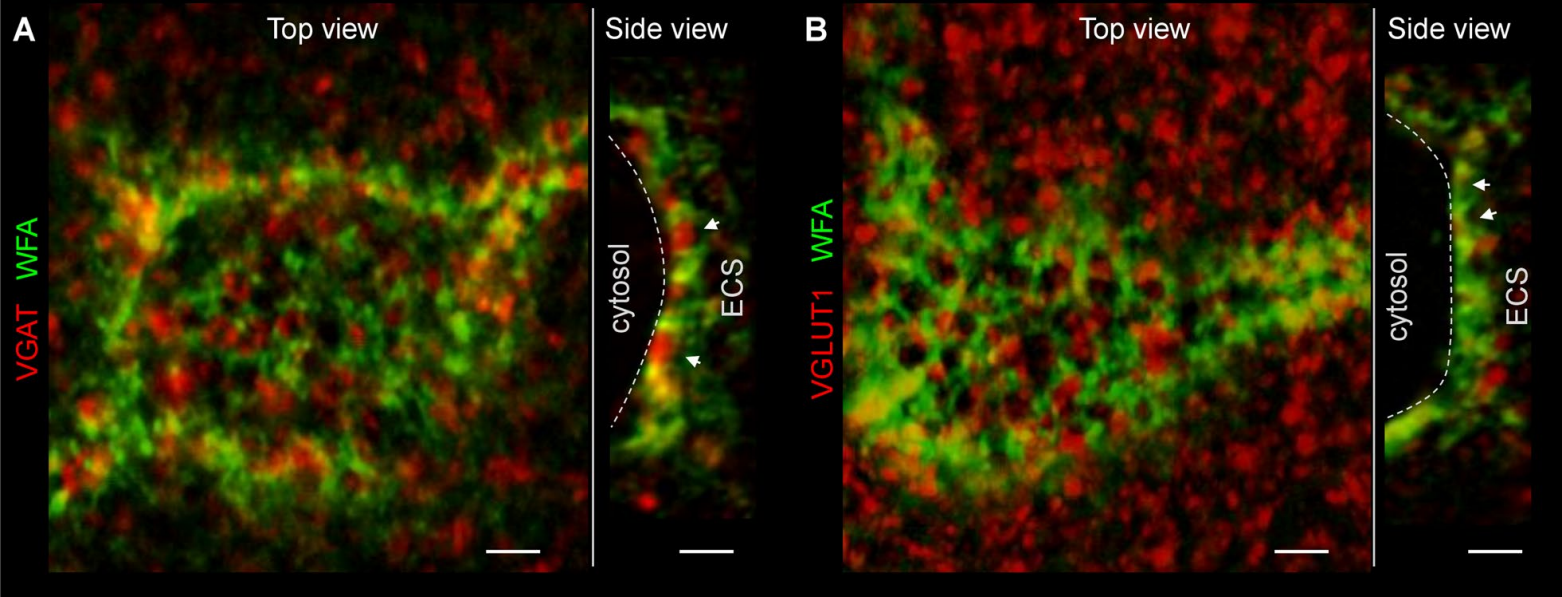
PNNs and their perforating synapses visualized using confocal microscopy. Confocal microscopy of WFA-labeled neurons shows characteristic facet-like morphology of PNNs. PNN facets form synaptic pockets (arrowheads), within which presynaptic terminals contact the neuronal surface (dash line) and establish synapses. GABAergic (**A**) and glutamatergic (**B**) terminals are shown by vesicular GABA (VGAT, red) and glutamate (VGLUT1) transporter labeling, correspondingly. Arrowheads and dash lines indicate putative synaptic pockets and neuronal surfaces, correspondingly. Scale bars, 2 µm. ECS, extracellular space

PNNs are exceptionally stable mesh-like structures in the extracellular space (ECS) consisting of densely packed proteoglycans bound together by link proteins [9, 17]. Chondroitin sulfate carrying proteoglycans of PNNs repel axons [4, 66], and the perisomatic synapses on fast-spiking interneurons are established within the PNN facets forming synaptic pockets, which can be visualized with confocal microscopy (Fig. 3).

After stroke, PNNs undergo morphological changes beyond all-or-none degradation, and the understanding of their structural remodeling requires three-dimensional superresolution imaging [21, 59]. Here, we juxtaposed PNN morphology visualized by four cutting-edge 3D imaging methods: two-photon (2P) excitation, confocal, superresolution structured illumination (SR-SIM) and stimulated emission depletion (STED) microscopy (Additional file 4: Fig S3). Comparison between the four methods indicated that both SR-SIM and 3D STED imaging methods allow for precise reconstruction of PNN morphology, but confocal and 2P microscopy are not suitable for this task due to insufficient resolution.

The morphology of PNNs was analyzed using the quantitative approach that combines 3D superresolution fluorescence imaging and graph-based computational reconstruction. Graphs thus obtained reflect the morphology of PNNs and allow for the quantitative analysis of their structure. In comparison to our previous report [21], we significantly improved imaging resolution by adjusting the refractive index of stained tissues (also known as tissue clearing), using fluorophores with longer emission wavelengths, and applying additional microscopy techniques.

**Fig. 4:**
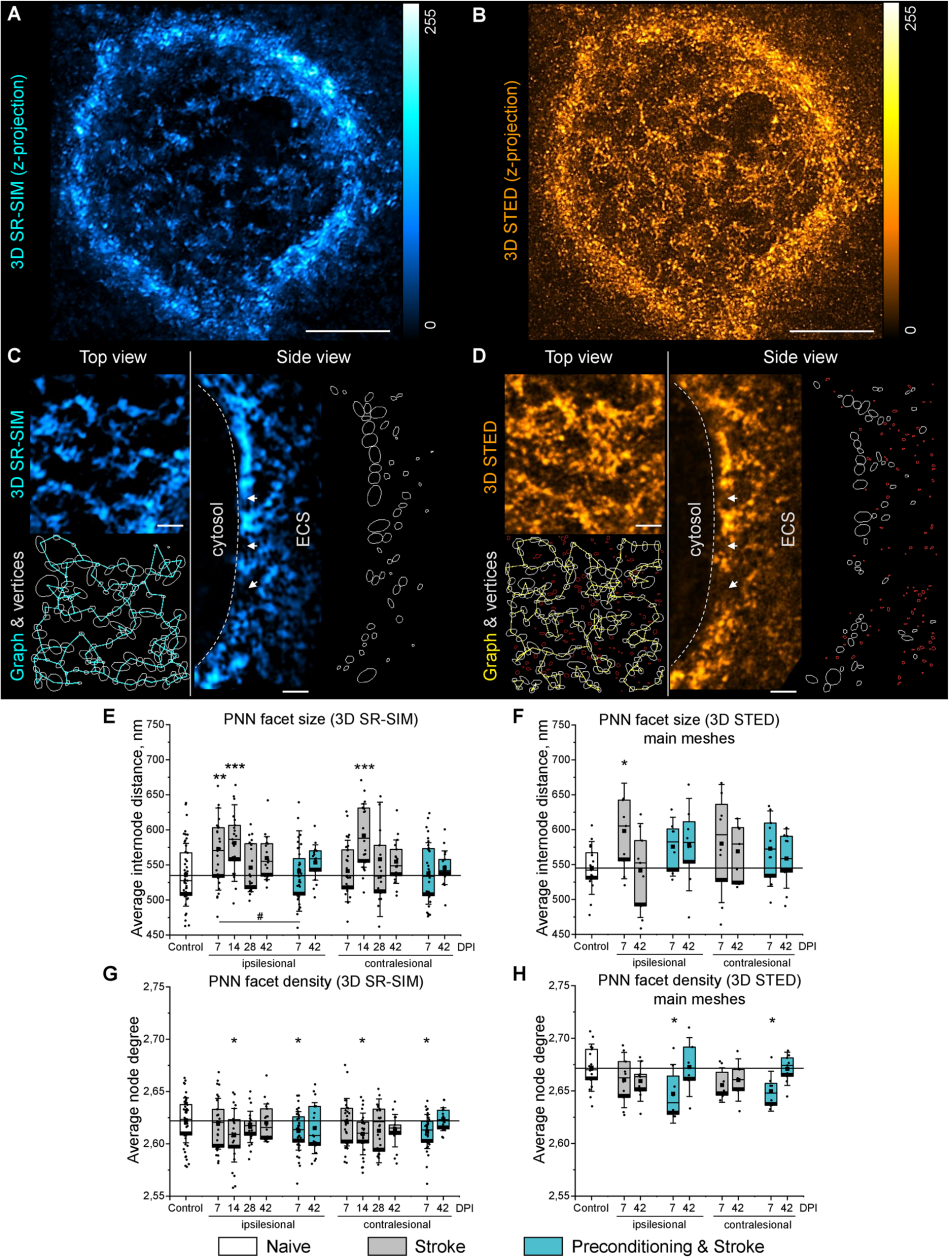
PNN morphology in the motor cortex L5 post stroke. **A**, **B** Maximum intensity z-projections show the morphology of the same PNN sequentially visualized using (**A**) SR-SIM and (**B**) STED microscopy. Scale bars, 5 µm. **C**, **D** High magnification single plane images, mesh vertices, and reconstructed graphs are shown for the same region visualized using (**C**) SR-SIM and (**D**) STED microscopy. Arrowheads and dash lines indicate putative synaptic pockets and neuronal surface, correspondingly. White outlines show PNN vertices, and red outlines are interstitial particles. Scale bars, 1 µm. **E**, **F** PNN facet size was quantified as average internode distance. **G**, **H** PNN facet density was quantified as average node degree. Graphs are box plots with data as dots, means as squares, medians as lines, interquartile ranges as boxes and SD as whiskers. Asterisks and hashes denote significant differences with the control and stroke groups, correspondingly, as indicated by two-way ANOVA and t-tests (*^,#^p < 0.05, **p < 0.01, ***p < 0.001), (**E**, **G**) n = 7, (**F**, **H**) n = 5. DPI, days post ischemia; SR-SIM, superresolution structured illumination microscopy; STED, stimulated emission depletion; ESC, extracellular space

To compare PNN morphology resolved by SR-SIM and 3D STED, we sequentially visualized the same PNN-coated neuron with the two methods (Fig. 4A, B) and aligned the obtained 3D image stacks based on the intensity maxima positions. Both methods revealed highly similar PNN structures, and both major vertices of the meshes and putative synaptic pockets were found at the same positions (Fig. 4C, D). Regions of interest showing putative synaptic pockets are magnified in Additional file 4: Fig S4.

Due to superior lateral resolution, 3D STED microscopy detected multiple smaller vertices in addition to those detected by SR-SIM (Fig. 4D). These intensity maxima were predominantly observed at a distance more than 1 µm from the neuronal surface and seldomly associated with synaptic pockets. We therefore concluded that these particles represent less condensed interstitial brain matrix and not PNNs. The small particles were filtered for PNN graph reconstruction, based on the cutoff defined by the Gaussian mixture model showing the bimodal distribution of WFA-labeled particle volumes measured by STED microscopy (Additional file 4: Fig S5A, B).

### Stroke induces transient loosening of PNNs in both hemispheres

We quantified morphological changes in motor cortical L5 PNNs (regions of interest are shown in Additional file 4: Fig S1) post stroke by measuring internode distances and node degrees (that is, the number of neighbors connected to a vertex) of the graphs reconstructing the organization of WFA-labeled meshes (Fig. 4E–H). Average internode distance (*L)* indicates PNN facet size, and average node degree (*D*) reflects facet density.

In mice exposed to stroke only, the size of PNN facets measured with SR-SIM (Fig. 4E) was significantly increased in the ipsilesional motor cortex at 7 DPI (*L* = 573 ± 59 nm) and 14 DPI (*L* = 581 ± 55 nm), and in the contralesional hemisphere at 14 DPI (*L* = 591 ± 45 nm), compared with the control (*L* = 536 ± 45 nm). At 28 and 42 DPI, PNN facet size decreased back to control levels. Inflammatory preconditioning minimized the observed effect, and PNN facet size was not different from the control at 7 and 42 DPI in mice exposed to preconditioning and stroke. We observed only a minor decrease in the density of PNN facets (Fig. 4G) at 14 DPI in the stroke group and at 7 DPI in the preconditioning and stroke group in both ipsilesional and contralesional hemispheres. The average size of the PNN nodes was not different between the groups (Additional file 4: Fig S6A).

The results obtained using SR-SIM were confirmed by 3D STED microscopy. The size of PNN facets measured with 3D STED was increased in the ipsilesional motor cortex at 7 DPI (*L* = 598 ± 60 nm versus *L* = 545 ± 33 nm in control) in mice exposed to stroke only (Fig. 4F). At 42 DPI, PNN facet size decreased back to control levels, and the size of the PNN nodes increased (Additional file 4: Fig. S6B), indicating condensation of ECM material. In mice with induced stroke tolerance, the facet size was not affected at 7 and 42 DPI, but we observed a minor decrease in the facet density at 7 DPI in both ipsilesional and contralesional hemispheres (Fig. 4H). In the interstitial matrix, we observed no alterations induced by stroke (Additional file 4: Fig. S5C-E).

### Transient PNN loosening associates with bilateral synaptic remodeling post stoke

**Fig. 5:**
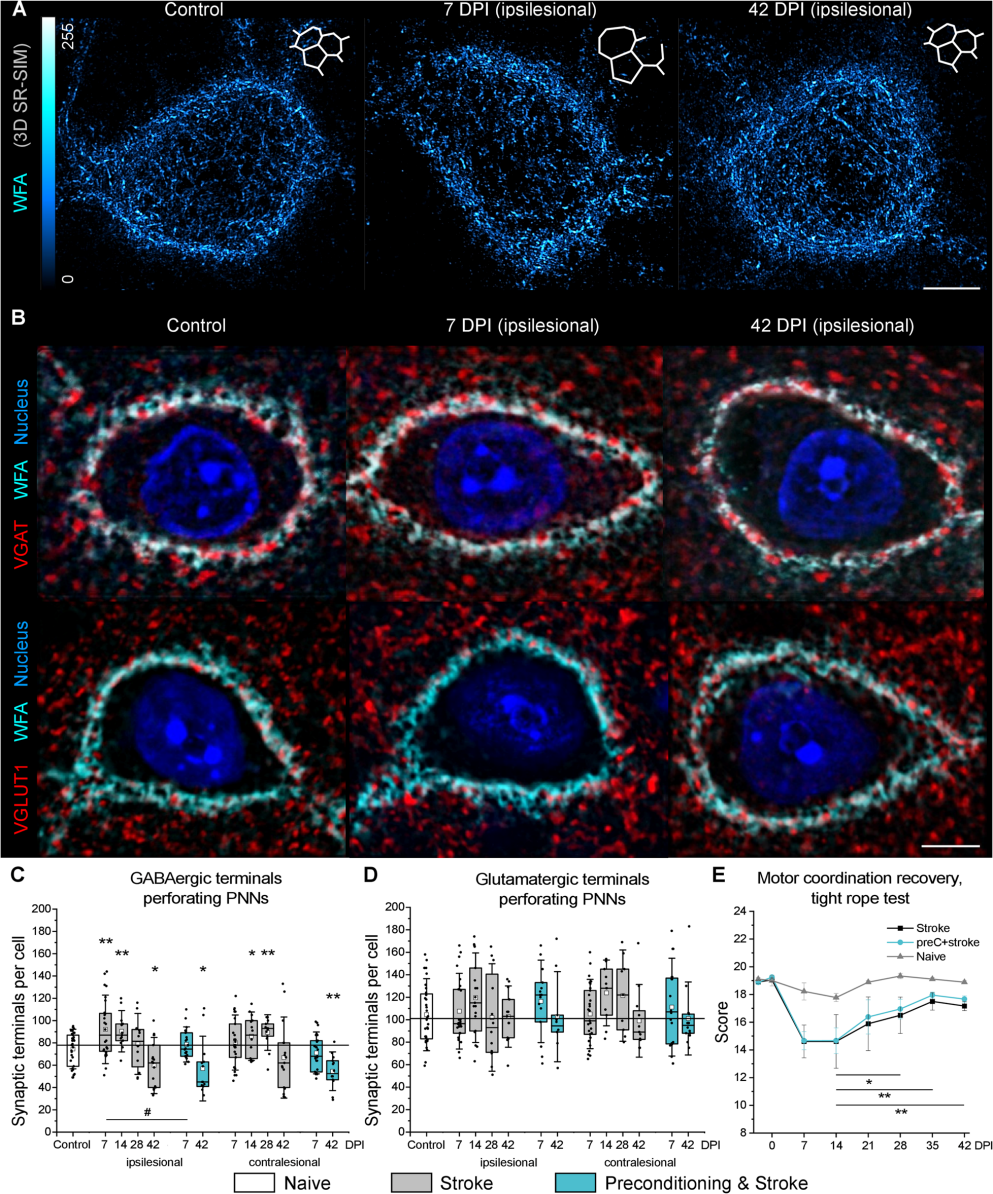
Coherent remodeling of PNNs and their perforating synaptic terminals precedes motor coordination recovery after stroke. **A** Representative maximum intensity z-projections show transient loosening of motor cortical PNNs after stroke detected by SR-SIM. Inlets show artistic representations of PNN facets. **B** Single-plane confocal images show representative immunolabeling of GABAergic axonal terminals expressing VGAT (red) and glutamatergic axonal terminals expressing VGLUT1 (red). PNNs were labeled with WFA (cyan), nuclei are shown in blue. Scale bars, 5 µm. **C**, **D** Quantifications show the number of GABAergic (**C**) and glutamatergic (**D**) terminals perforating PNNs. Graphs are box plots with data as dots, means as squares, medians as lines, interquartile ranges as boxes and whiskers showing SD. Asterisks and hashes denote significant differences with the control and stroke groups, correspondingly, as indicated by two-way ANOVA and t-tests (*^,#^p < 0.05, **p < 0.01), n = 7. **E** Motor coordination recovery measured by tight rope test. Data are mean ± s.e.m. n = 7. DPI, days post ischemia; ns, not significant

Because PNNs have a major impact on synaptic plasticity [27], we further explored whether the transient loosening of PNN facets post stroke (Fig. 5A) associates with synaptic remodeling. The density of GABA- and glutamatergic terminals perforating PNNs was quantified in ROIs containing a single PNN coated motor cortical neuron (Fig. 5B).

In healthy brains, motor cortical L5 interneurons received 75 ± 15 (mean ± SD) GABAergic inputs from their network partners, as indicated by the quantifications of VGAT terminals perforating PNNs (Fig. 5C). In mice exposed to stroke only, the number of GABAergic terminals perforating PNNs increased at 7 and 14 DPI (92 ± 26 and 90 ± 15 inputs per cell) in the ipsilesional motor cortex but was decreased at 42 DPI (60 ± 21 inputs per cell). In the contralesional motor cortex, interneurons received more GABAergic inputs at 14 and 28 DPI (86 ± 18 and 89 ± 13 inputs per cell) but the number of VGAT terminals decreased back to control levels at 42 DPI. In mice treated with inflammatory preconditioning, the number of GABAergic terminals decreased at 42 DPI (57 ± 24 inputs per cell) bilaterally but did not differ with the control at 7 DPI.

Motor cortical L5 interneurons in healthy brains received 104 ± 27 (mean ± SD) glutamatergic inputs from their local network partners, as indicated by the quantifications of VGLUT1 terminals perforating PNNs (Fig. 5D), and their number was not significantly altered post stoke.

### Motor recovery follows motor cortical tissue remodeling post stroke

We analyzed neurological recovery after stroke using Clark’s score for general and focal deficits and tight rope test for measuring motor performance. Neurological deficits (Additional file 4: Fig S7) manifested in the acute phase and decreased during the first week post stroke. Motor activity and coordination measured by the tight rope test (Fig. 5E) were decreased at 7 DPI. Starting 28 DPI, we observed a gradual recovery of coordinated movements. Motor coordination was significantly restored by 42 DPI. We observed no significant influence of immune preconditioning on the motor performance recovery after stroke. These data indicate that the transient loosening of PNNs and synaptic remodeling in the motor cortex L5 precede the recovery of coordinated motor activity post stroke.

### Surface contact between neurons and microglia/macrophages increases post stroke

**Fig. 6:**
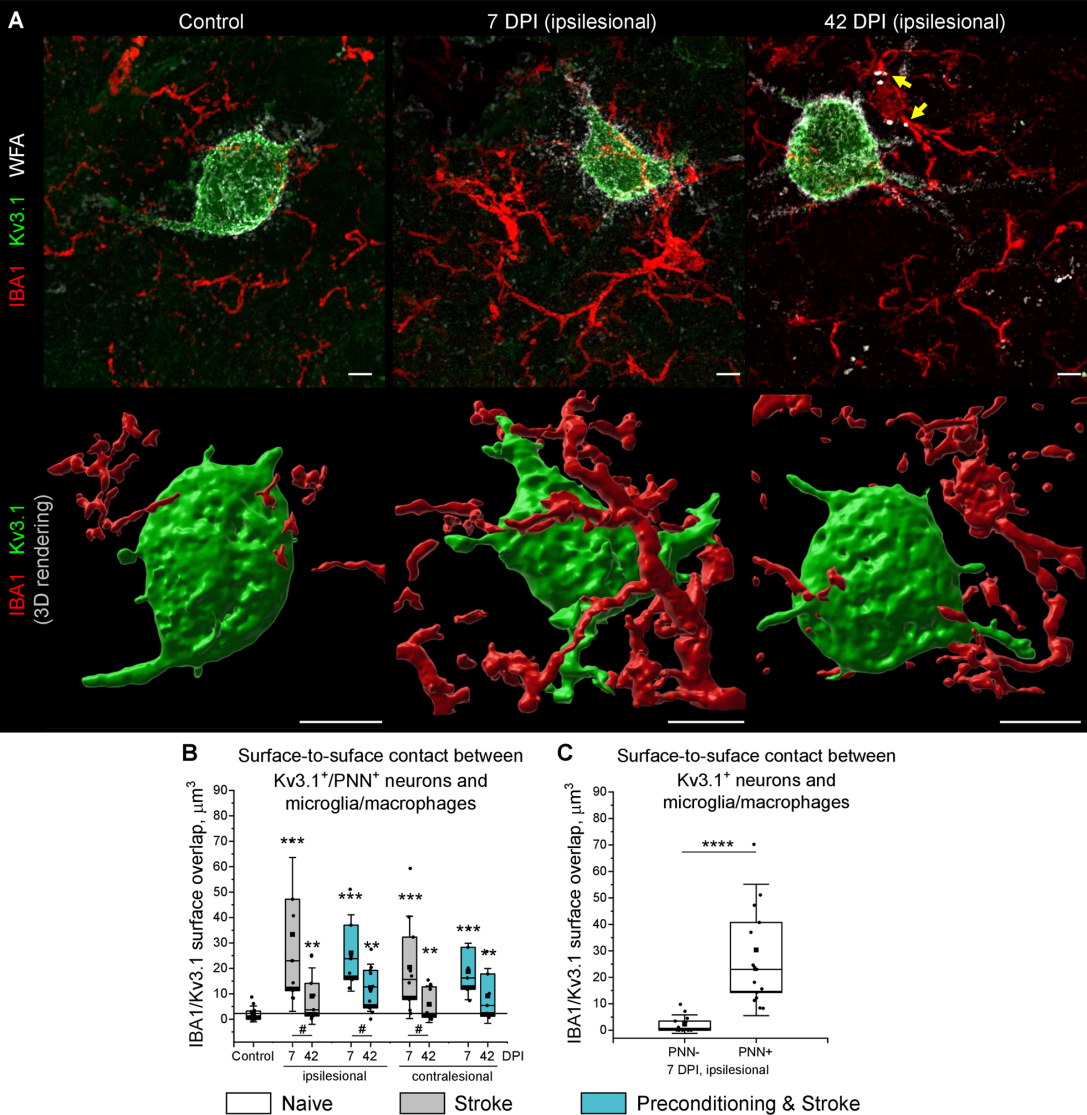
Surface contacts between interneurons and microglia/macrophages post stroke. **A** Confocal Z-projections show representative IBA1 (red), Kv3.1 (green) and WFA (white) immunolabeling in the motor cortex L5. Yellow arrowheads highlight speckles of WFA-labeled material inside IBA1-labelled cell bodies at 42 DPI. The corresponding 3D rendering of IBA1 and Kv3.1 surfaces is shown below. Scale bars, 5 µm. **B** Surface contact between microglia/macrophages and Kv3.1^+^/PNN^+^ cells. **C** Surface contact between microglia/macrophages and Kv3.1^+^ neurons with and without PNNs at 7 DPI. Graphs are box plots with data as dots, means as squares, medians as lines, interquartile ranges as boxes, and whiskers showing SD. Asterisks and hashes denote significant differences with the control and 7 DPI groups, correspondingly, as indicated by two-way ANOVA and t-tests (^#^p < 0.05, ***p < 0.001, ****p < 0.00001). n = 5. DPI, days post ischemia

In a healthy brain, microglia/macrophages establish direct contact with neuronal plasma membranes [16] and can promote synaptic plasticity by remodeling extracellular matrix [47]. In the fast-spiking PNN coated interneurons, the direct surface contact with microglia/macrophages should be difficult because of the inhibitory properties of incorporated proteoglycans [1, 20, 27]. However, the transient loosening of PNNs after stroke (Fig. 5A) may facilitate interneuron-microglia/macrophages interaction. We explored this possibility by quantifying the surface-to-surface contacts between microglia/macrophages labeled with IBA1 and the fast-spiking neurons expressing Kv3.1 channels (Fig. 6A) in the motor cortex L5 after stroke.

Under all experimental conditions that we investigated, multiple microglia/macrophage processes were observed in close proximity to neuronal membranes expressing Kv3.1, and at least two microglia/macrophage cell bodies were present within a 50 µm radius around the soma of every Kv3.1^+^ neuron. While in the healthy brains, the direct contacts between microglia/macrophages and Kv3.1^+^/PNN^+^ neurons were point-like and not numerous, the IBA1/Kv3.1 surface overlap strongly increased at 7 DPI in both ipsilesional and contralesional hemispheres (Fig. 6B). At 42 DPI, microglia/macrophage-interneuron contact surface decreased in comparison to 7 DPI but remained significantly larger than in control. In mice with inflammatory preconditioning, the IBA1/Kv3.1 surface overlap was increased similar to the stroke only group.

As demonstrated by 3D surface rendering (Fig. 6A), microglia/macrophages enwrapped significant parts of neuronal surfaces at 7 DPI. Interestingly, the extensive contacts between Kv3.1^+^ neuronal membranes and microglia/macrophage processes were observed only on Kv3.1^+^/PNN^+^ cells, indicating the high preference of microglia/macrophages to contacting PNN-coated neurons (Fig. 6C). In addition, we observed multiple WFA-labeled speckles inside IBA1-labeled cells at 42 DPI (Fig. 6A), suggesting that microglia/macrophages can phagocyte PNN components after stroke.

## Discussion

We herein demonstrate that the increased size and reduced density of PNN facets associate with synaptic reorganization preceding the recovery of motor coordination after stroke. Noteworthy, morphological changes in motor PNNs revealed by SR-SIM were confirmed using 3D STED microscopy. The coherent remodeling of PNN structure and GABAergic axonal terminals on motor cortical L5 interneurons suggests a novel mechanism of stroke recovery that involves ECM modulation in both ipsi- and contralesional hemispheres. During the subacute stroke phase at 7 DPI, PNN loosening correlates with the increasing number of perforating axonal terminals expressing VGAT, which agrees with increased GABAergic phasic activity during the first week post stroke [38]. In the chronic stroke phase at 42 DPI, PNN morphology returns back to normal, but the number of GABAergic inputs received by motor cortical L5 interneurons is significantly reduced. We hypothesize that these dynamic changes in motor cortical inhibitory connectivity arise from the tripartite interaction between PNNs, synapses, and microglia/macrophages.

PNNs cover the soma, proximal dendrites and initial axonal segments of the fast-spiking interneurons that express calcium binding protein PV and potassium channels Kv3.1 with fast activation and deactivation kinetics [35, 45]. By creating facet-like structures, PNNs compartmentalize neuronal surface and restrict synapse formation to the areas devoid of inhibitory CSPGs that repel axons [4]. The three-dimensional organization of PNN facets resembles wells that are approximately 1 µm deep, with neuronal plasma membrane at the bottom and opening towards the extracellular space. Because most of these wells are occupied by perforating axonal terminals, we here propose to call them synaptic pockets. After stroke, the increased size of synaptic pockets allows for new synapse formation. Recent findings show that synapses continuously wane and re-emerge in vivo and that GABAergic terminals are especially dynamic with about 60% of them retracting and returning within a few days [65]. We propose that the loosening of PNNs creates larger permissive regions supporting GABAergic synapse plasticity and results in increased GABAergic input to fast-spiking interneurons at 7 and 14 DPI. These new synapses are not stabilized in the long term though, which leads to the decreased number of VGAT terminals perforating PNNs at 42 DPI. Importantly, we observed PNN loosening and synaptic remodeling in both ipsi- and contralesional hemispheres, which suggests that ECM modulation supports bilateral rewiring of brain circuits after stroke.

The removal of new GABAergic synapses that are established during the post-acute stroke phase is likely mediated by activated microglia/macrophages. Recent evidence indicates that microglia/macrophages can selectively sculpt inhibitory connectivity [24] by eliminating presynaptic terminals using a phagocytic mechanism known as trogocytosis [68]. Here, we observed that every PNN-coated fast-spiking interneuron in the motor cortex L5 is always adjacent to a few highly ramified microglia/macrophages. Our data shows that although increased IBA1 immunoreactivity persists in the chronic stroke phase, the surface-to-surface contact between interneurons and microglia/macrophages is more extensive at 7 DPI than at 42 DPI. Interestingly, the surface of microglia/macrophage-interneuron contacts on PNN^+^ cells is 30 times larger than on PNN^−^ neurons at 7DPI, indicating the high microglia/macrophage preference for enwrapping PNN-coated neurons in the ischemic brain.

At 42 DPI, we observed speckles of WFA-labeled substance within IBA1-labeled cell bodies, suggesting that microglia/macrophage activation contributes to the PNN loosening observed at 7 DPI. Notably, activated microglia/macrophages facilitate the loss of PNNs in rodent models of Alzheimer’s [15] and Huntington’s [14] diseases. We suppose that after stroke, microglia/macrophages attenuate plasticity inhibiting PNN properties in the early post-acute phase, allowing for the formation of additional GABAergic synapses on motor cortical L5 interneurons. Noteworthy, inhibitory interneurons expressing parvalbumin and PNNs play a major role in regulating activity and synchronization in neuronal networks [25]. The increased inhibitory input to inhibitory interneurons can switch the excitation-inhibition balance in motor cortical microcircuits towards excitation, thereby promoting motor coordination recovery that we observed starting 28 DPI. During the chronic stroke phase, PNNs recover completely and restrict new synapse formation, while the non-stabilized inputs are removed by microglia/macrophages. Thus, PNNs and microglia/macrophages negatively regulate inhibitory input to inhibitory interneurons, which can limit functional recovery in the chronic stroke phase.

Admittedly, our hypothesis is based on correlations and needs further validation using intravital superresolution imaging. Here, we revealed the transient remodeling of PNNs in the motor cortical L5 after stroke using SR-SIM and confirmed this effect using STED microscopy. While SR-SIM can be applied for multi-label imaging in relatively large volumes, it requires sophisticated computational processing that may generate artifacts [36]. STED microscopy has superior resolution compared to SR-SIM and does not generate any processing-related artifacts [6]. However, STED microscopy is more challenging to perform in multi-color modes and commonly uses short-distance objectives that make 3D imaging in large volumes limited.

In this work, we visualized and quantified synaptic terminals and microglia/macrophage-neuron contacts using high-resolution confocal microscopy. Nevertheless, our measurements relied on immunohistochemical procedures incompatible with intravital imaging. In the future, the tripartite interaction between PNN, presynaptic terminals, and microglia/macrophages post stroke can be verified using reversibly switchable fluorescent proteins and multi-label in vivo STED microscopy [69]. In comparison to our previous work [21], here we improved the method for PNN morphology reconstruction by significantly improving imaging resolution and optimizing the graph computation algorithm. As a result, the internode distances that we measured in this study are twice smaller than detected with our previous method. Improved resolution of imaging allowed us to detect the previously unnoticed increase of PNN facet size after stroke. With the earlier method, we detected a slight decrease in average internode distance, which is misleading and due to the merging of the adjacent PNN nodes because of insufficient resolution. Similar to our previous study, here we detected a decrease in the average degree of PNN nodes after stroke. However, the correct connectivity degrees for PNN nodes are between 2.5 and 2.8 as we show here, and not between 2.5 and 4 as we previously reported due to insufficient imaging resolution. Conclusively, superior microscopy techniques and cross-validation of imaging approaches in this study allowed for the detection of correct parameters of PNN morphology. Using the improved methodology, we revealed that the structural alterations in PNNs are associated with synaptic remodeling and functional recovery post stroke.

The superior resolution of STED microscopy and exceptional photostability of WFA labeling allowed for the detection of interstitial ECM particles that did not associate with PNNs. In rodent models of learning and memory formation, the interstitial matrix regulates axonal sprouting and synaptic input density, while PNNs control the number of synaptic spines and receptor mobility [26]. In the stroke model we used herein, the interstitial matrix density was not affected, and the dynamic modulation of GABAergic input density on motor cortical L5 interneurons was associated with structural rearrangements in PNNs. This evidence indicates that the role of cortical PNNs in post stroke recovery differs from the memory-related function of hippocampal PNNs.

Despite the prominent changes in PNN morphology, the number of glutamatergic inputs on the fast-spiking motor cortical L5 interneurons is not affected post stroke. Noteworthy, the glutamatergic terminals that we detected here by VGLUT1 immunolabeling represent the local excitatory input within motor cortical microcircuits and not the thalamocortical afferents [28]. The higher stability of glutamatergic inputs on interneurons post stroke may involve additional mechanisms independent of PNNs [5, 64], which agrees with our recent study showing that the depletion of ECM primarily affects inhibitory and not excitatory synapses [19].

Here, we compared PNN morphology alterations, synaptic remodeling, and reactive gliosis after focal cerebral ischemia in mice with and without induced stroke tolerance. Inflammatory preconditioning via systemic injection of LPS has been shown to reduce the severity of stroke in animal models [44, 60]. Our data indicate that exposure to inflammatory stress before stroke reduces infarct volume, attenuates PNN loosening, and prevents synaptic alterations during the subacute stroke phase. Although based on our data the delayed stroke progression in pre-conditioned mice cannot be completely excluded, the dynamics of motor coordination recovery were similar in animals with and without stroke tolerance, making such a scenario unlikely. Hence, the extent of structural changes in PNNs and their perforating GABAergic synapses depends predominantly on the severity of ischemic injury.

In the chronic phase, GABAergic input is similarly reduced in mice with and without induced stroke tolerance. Inflammatory preconditioning does not reduce brain atrophy and even increases microglia/macrophage reactivity at 42 DPI. We also observed no significant effect of inflammatory preconditioning on motor recovery in the chronic stroke phase, which calls into question the translational value of this approach for improving long-term recovery after stroke.

Our results suggest that the alternating morphology and integrity of PNNs in the motor cortex can be therapeutically utilized for promoting neurological recovery in the chronic stroke phase. While the intrinsic brain remodeling post stroke involves only transient loosening of PNNs, prolonging the partial PNN degradation during the post-acute period might extend the opening neuroplasticity window into the chronic stroke phase, which might be achieved by modulating microglial phagocytic activity [62]. In addition, modulation of CSPG sulfation is a promising target for improving stroke recovery. The plasticity-inhibiting properties of CSPGs depend on the ratio between 4-sulfated and 6-sulfated disaccharides [31, 66]. Outgrowing axons avoid 4-sulfated CSPGs, and their selective cleavage with arylsulfatase B has been proposed for promoting nerve regeneration [51].

We are convinced that the development of novel restorative stroke therapies necessitates exploring the translational potential of modulating key components of ECM-mediated signaling in the recovering brain. Few experimental studies so far explored the possibility of selectively modulating ECM components for promoting stroke recovery. This is surprising, since experimental models and pharmacological molecules exist that allow for manipulating the ECM components and detecting neurological recovery responses in a clinically meaningful way. Pharmacological treatments targeting the ECM have shown promising outcomes in clinical trials in cancer [70] and atherosclerosis [71], but they have not been systematically studied after stroke. Emerging paradigms in stroke research emphasize the importance of targeting the correct tissue compartments at different time points post stroke [43]. From this new perspective, perineuronal ECM represents a novel and promising target for promoting neuroplasticity and stroke recovery.

## Conclusions

Our data shows that post-stroke motor recovery is preceded by transient reorganization of PNNs and their perforating synapses in the motor cortical L5, revealing a novel mechanism of neuroplasticity involving the tripartite interaction between ECM, neurons, and microglia/macrophages in the ischemic brain. These findings challenge the traditional understanding of stroke recovery mechanisms and suggest that targeted interventions modulating ECM components may revolutionize stroke treatment.

### Supplementary Information

**Additional file 1. **Supplementary ImageJ macros for synapse quantification.

**Additional file 2. **Supplementary MATLAB code for PNN morphology quantification.

**Additional file 3. **Power calculations.

**Additional file 4: Figure S1.** Regions of interest selected for immunohistochemical analysis. In each animal, two adjacent coronal sections at bregma level were analyzed. Microscopic analysis was performed in four regions of interest (ROIs) per section, as indicated by black squares on the schematic. **Figure S2.** Expression of Kv3.1 and parvalbumin in the motor cortical PNN^+^ neurons. (A) Cell density of neurons expressing PNNs. (B) Percentage of PNN^+^ neurons expressing Kv3.1. (C) Percentage of PNN^+^ neurons expressing PV. (D, E) Cell density of neurons expressing PV (D) and Kv3.1 (E). Graphs are bar plots showing mean ± SD and data as dots. Asterisks and hashes denote significant differences with the control group, as indicated by two-way ANOVA and t-tests (*p < 0.05, **p < 0.01), n = 7. DPI, days post ischemia; PV, parvalbumin. **Figure S3.** PNN morphology analysis using 2P, confocal, SIM, and STED microscopy. (A) PNNs in the motor cortex L5 (control brains) were labeled with biotinylated WFA and streptavidin conjugated to Atto 490 (2P microscopy) or Star RED (confocal, SR-SIM and 3D STED microscopy) fluorophores. Images are maximum intensity z-projections. Scale bars, 10 µm. (B) Lateral (Dxy) and axial (Dz) imaging resolution was estimated as the full width at half-maximum (FWHM) using sub-resolution fluorescent beads (Ø 100 nm and Ø 40 nm for STED) embedded in the stained tissue. Scale bars, 500 nm. Notably, the lateral resolution of confocal microscopy (Dxy = 217 ± 15 nm) was very close to the diffraction limit (d = λ/2NA = 633/2.92 = 216.8 nm. (C) PNN morphology was reconstructed as graphs with nodes positioned at local fluorescence intensity maxima and edges generated by a non-redundant nearest neighbor search algorithm.). Both SR-SIM and 3D STED, but not 2P and confocal imaging allowed for precise reconstruction of PNN morphology using graphs, the mathematical constructs designed for topological analysis. (D) Histograms show internode distance distributions for the single PNNs shown in (A) and (C). 2P, two-photon excitation; SR-SIM, superresolution structured illumination microscopy; STED, stimulated emission depletion; D_xy_, lateral resolution; D_z_, axial resolution. **Figure S4.** Structure of a putative synaptic pocket. High magnification single plane images, mesh vertices, and the reconstructed graphs are shown for the same region visualized using (A) SR-SIM and (B) STED microscopy. Scale bars, 500 nm. **Figure S5.** STED microscopy detects both PNN vertices and interstitial matrix particles. (A) Histograms show the distribution of WFA-labeled particle volumes measured with SR-SIM and STED microscopy in control mice (n = 5). Bin size, 0.01 µm^3^. (B) Gaussian mixture model indicates the bimodal distribution of PNN vertex volumes measured by STED microscopy. The 0.03 µm^3^ cutoff was chosen to filter the small vertices not associating with PNNs. (C) High magnification single plane STED image, PNN vertices, interstitial matrix particles, and the reconstructed interstitial matrix graph are shown. Scale bars, 500 nm. Interparticle distance (D) and meshwork density (E) quantifications indicate no significant alterations in the interstitial matrix post stroke. Graphs are box plots with data as dots, means as squares, medians as lines, interquartile ranges as boxes and whiskers showing SD. n = 5. DPI, days post ischemia. **Figure S6.** Size of PNN nodes post stroke. The size of PNN nodes was measured with superresolution (A) SR-SIM and (B) 3D STED microscopy as the volume of ellipsoids. Graphs are box plots with data as dots, means as squares, medians as lines, interquartile ranges as boxes and whiskers showing SD. Asterisks denote significant differences with the control group, as indicated by two-way ANOVA and t-tests (***p < 0.001), n = 5. DPI, days post ischemia. **Figure S7.** Neurological deficits post stroke. (A) Clark’s general deficits scoring. (B) Clark’s focal deficits scoring. Data are mean ± s.e.m. n = 7. DPI, days post ischemia.

## Data Availability

The datasets generated during this study are available in the Dryad repository: https://datadryad.org/stash/share/0QhvpPe4RvVi3d5NAhRz727RZ8KfaL9eAGsPrCB3WRE.

## Abbreviations

2P: Two-photon
AA: Atrophic area
AV: Atrophy volume
CCA: Common carotid artery
CSPG: Chondroitin sulfate proteoglycan
DPI: Days post ischemia
DPSS: Double pumped solid state
ECM: Extracellular matrix
ECS: Extracellular space
FWHM: Full width at half-maximum
GABA: γ-Aminobutyric acid
GFAP: Glial acidic fibrillary protein
IA: Infarcted area
i.v.: Intravenously
LDF: Laser Doppler flow
LPS: Lipopolysaccharide
LSM: Laser scanning microscopy
MCA: Middle cerebral artery
PBS: Phosphate buffered saline
PFA: Paraformaldehyde
PNN: Perineuronal net
PV: Parvalbumin
ROI: Region of interest
SR: Superresolution
SIM: Structured illumination microscopy
STED: Stimulated emission depletion
tMCAO: Temporary middle cerebral artery occlusion
TDE: Thiodiethanol
VGAT: Vesicular GABA transporter
VGLUT1: Vesicular glutamate transporter 1
WFA: *Wisteria floribunda* Agglutinin
