# Supplementary material for: Structural changes in perineuronal nets and their perforating GABAergic synapses precede motor coordination recovery post stroke
Source: J Biomed Sci. 2023 Sep 1;30:76. doi: 10.1186/s12929-023-00971-x (PMC10474719; doi:10.1186/s12929-023-00971-x)
Supplement: Supplementary file 4 — Additional file 4: Figure S1. Regions of interest selected for immunohistochemical analysis. In each animal, two adjacent coronal sections at bregma level were analyzed. Microscopic analysis was performed in four regions of interest (ROIs) per section, as indicated by black squares on the schematic. Figure S2. Expression of Kv3.1 and parvalbumin in the motor cortical PNN+ neurons. (A) Cell density of neurons expressing PNNs. (B) Percentage of PNN+ neurons expressing Kv3.1. (C) Percentage of PNN+ neurons expressing PV. (D, E) Cell density of neurons expressing PV (D) and Kv3.1 (E). Graphs are bar plots showing mean ± SD and data as dots. Asterisks and hashes denote significant differences with the control group, as indicated by two-way ANOVA and t-tests (*p < 0.05, **p < 0.01), n = 7. DPI, days post ischemia; PV, parvalbumin. Figure S3. PNN morphology analysis using 2P, confocal, SIM, and STED microscopy. (A) PNNs in the motor cortex L5 (control brains) were labeled with biotinylated WFA and streptavidin conjugated to Atto 490 (2P microscopy) or Star RED (confocal, SR-SIM and 3D STED microscopy) fluorophores. Images are maximum intensity z-projections. Scale bars, 10 µm. (B) Lateral (Dxy) and axial (Dz) imaging resolution was estimated as the full width at half-maximum (FWHM) using sub-resolution fluorescent beads (Ø 100 nm and Ø 40 nm for STED) embedded in the stained tissue. Scale bars, 500 nm. Notably, the lateral resolution of confocal microscopy (Dxy = 217 ± 15 nm) was very close to the diffraction limit (d = λ/2NA = 633/2.92 = 216.8 nm. (C) PNN morphology was reconstructed as graphs with nodes positioned at local fluorescence intensity maxima and edges generated by a non-redundant nearest neighbor search algorithm.). Both SR-SIM and 3D STED, but not 2P and confocal imaging allowed for precise reconstruction of PNN morphology using graphs, the mathematical constructs designed for topological analysis. (D) Histograms show internode distance distributions for the [file 12929_2023_971_MOESM4_ESM.docx]

**Supporting information**

**
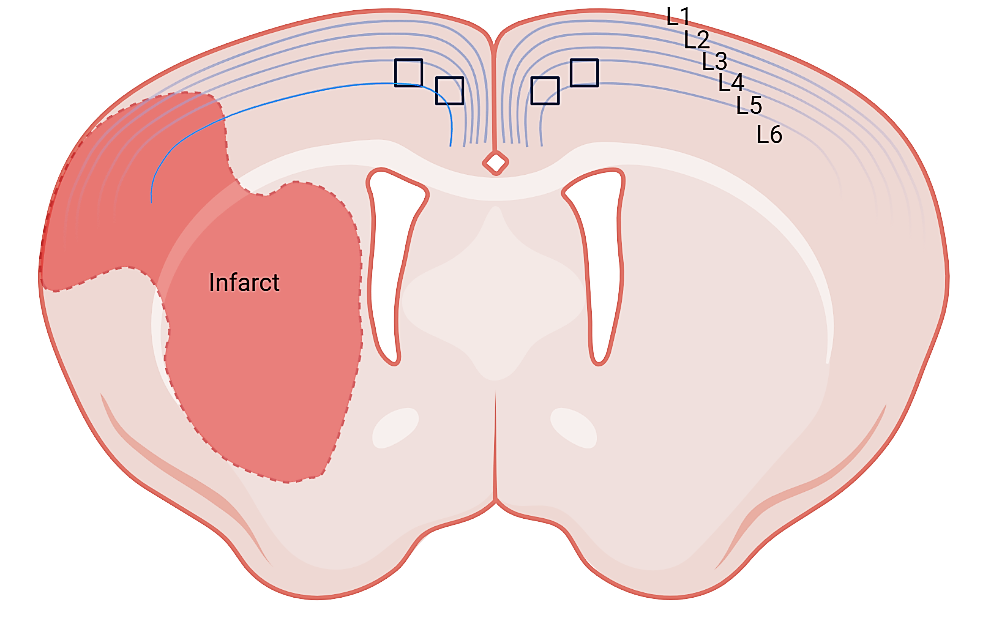
**

**Supplementary Figure S1. Regions of interest selected for immunohistochemical analysis.** In each animal, two adjacent coronal sections at bregma level were analyzed. Microscopic analysis was performed in four regions of interest (ROIs) per section, as indicated by black squares on the schematic.


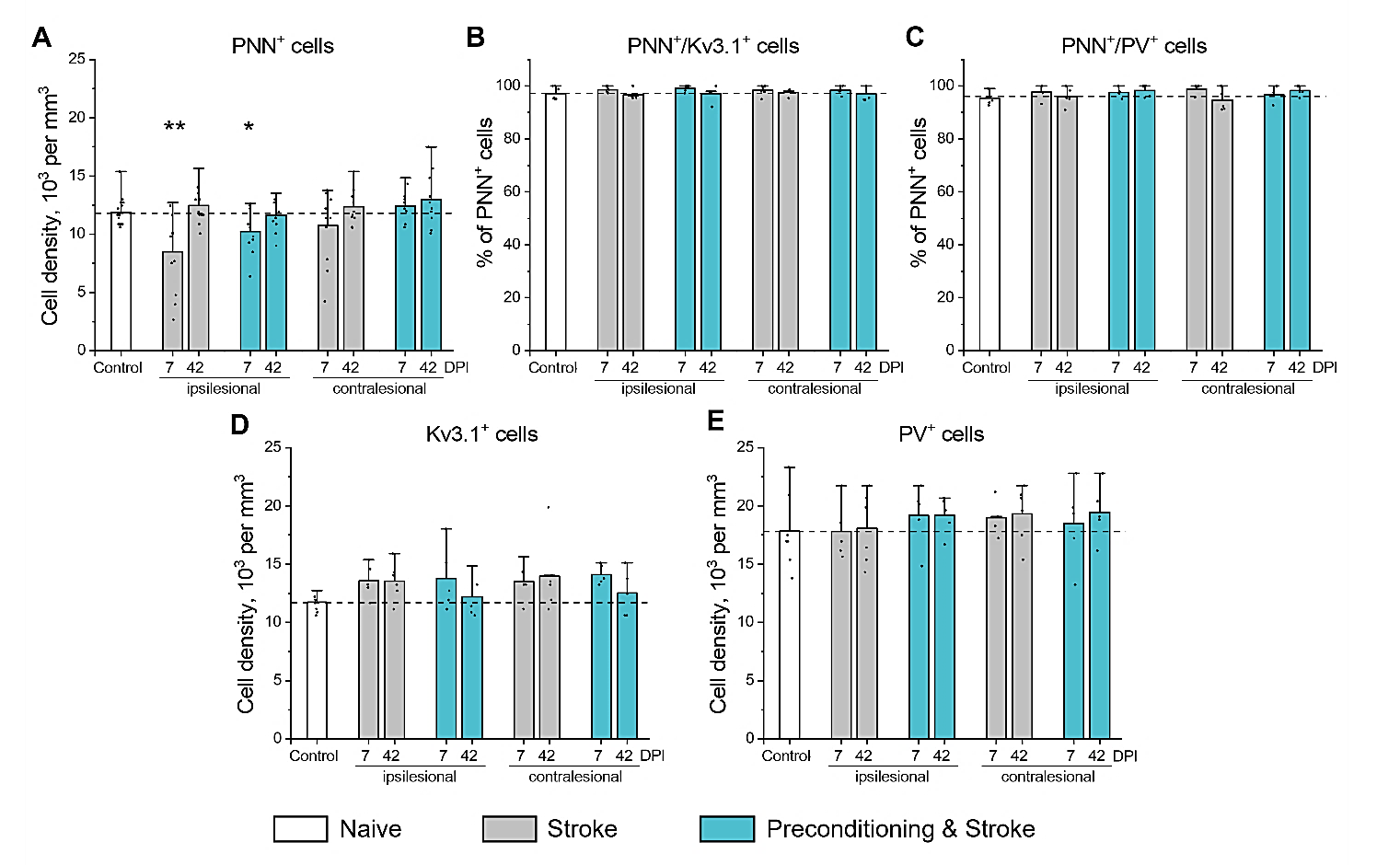
**Supplementary Figure S2. Expression of Kv3.1 and parvalbumin in the motor cortical PNN^+^ neurons.** (**A**) Cell density of neurons expressing PNNs. (**B**) Percentage of PNN^+^ neurons expressing Kv3.1. (**C**) Percentage of PNN^+^ neurons expressing PV. (**D**, **E**) Cell density of neurons expressing PV (**D**) and Kv3.1 (**E**). Graphs are bar plots showing mean±SD and data as dots. Asterisks and hashes denote significant differences with the control group, as indicated by two-way ANOVA and t-tests (*p < 0.05, **p < 0.01), n = 7. DPI, days post ischemia; PV, parvalbumin.


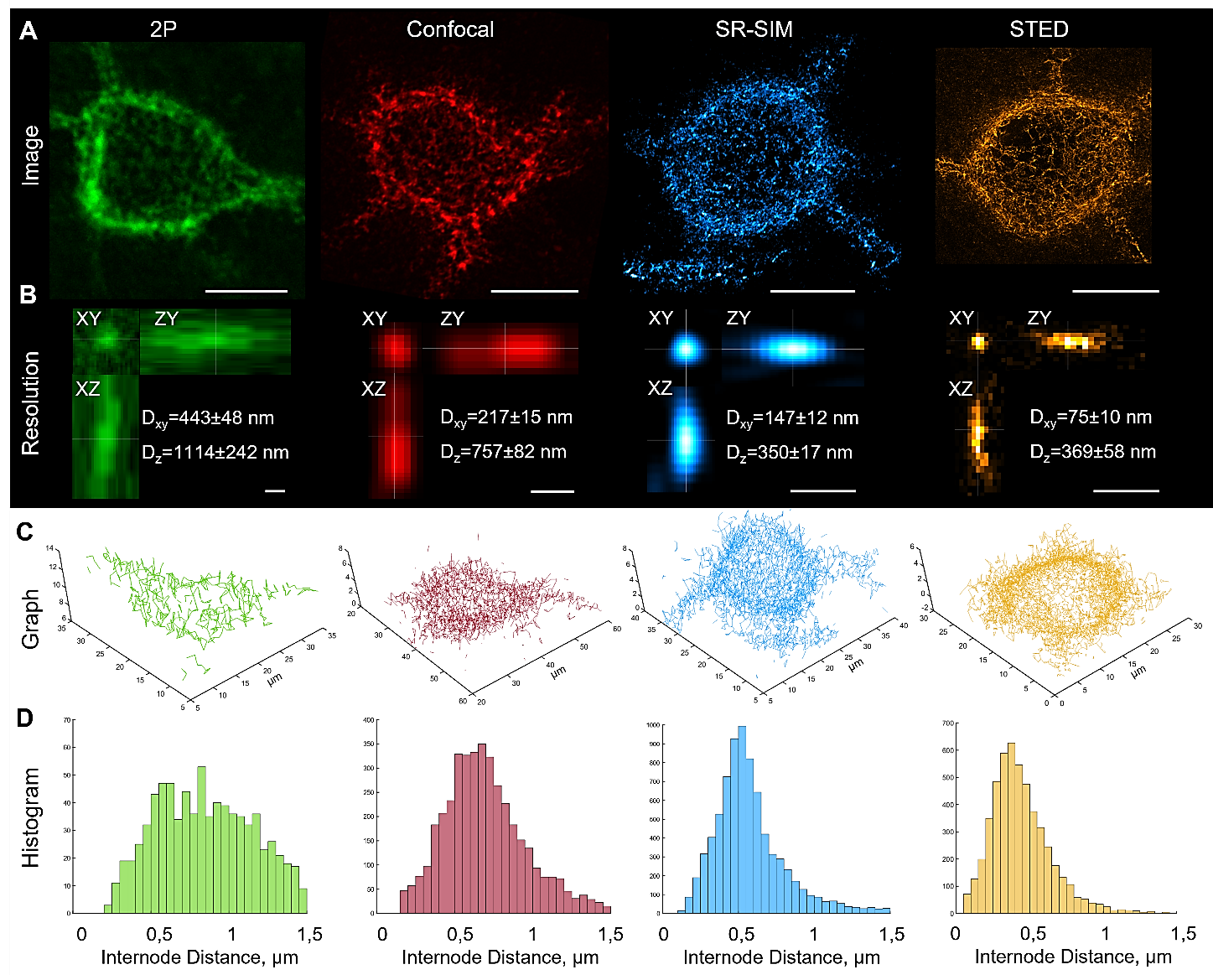


**Figure S3. PNN morphology analysis using 2P, confocal, SIM, and STED microscopy.**

(**A**) PNNs in the motor cortex L5 (control brains) were labeled with biotinylated WFA and streptavidin conjugated to Atto 490 (2P microscopy) or Star RED (confocal, SR-SIM and 3D STED microscopy) fluorophores. Images are maximum intensity z-projections. Scale bars, 10 µm. (**B**) Lateral (Dxy) and axial (Dz) imaging resolution was estimated as the full width at half-maximum (FWHM) using sub-resolution fluorescent beads (Ø 100 nm and Ø 40 nm for STED) embedded in the stained tissue. Scale bars, 500 nm. Notably, the lateral resolution of confocal microscopy (Dxy = 217±15 nm) was very close to the diffraction limit (d = λ/2NA = 633/2.92 = 216.8 nm. (**C**) PNN morphology was reconstructed as graphs with nodes positioned at local fluorescence intensity maxima and edges generated by a non-redundant nearest neighbor search algorithm. ). Both SR-SIM and 3D STED, but not 2P and confocal imaging allowed for precise reconstruction of PNN morphology using graphs, the mathematical constructs designed for topological analysis. (**D**) Histograms show internode distance distributions for the single PNNs shown in (**A)** and (**C**). 2P, two-photon excitation; SR-SIM, superresolution structured illumination microscopy; STED, stimulated emission depletion; D_xy_, lateral resolution; D_z_, axial resolution.


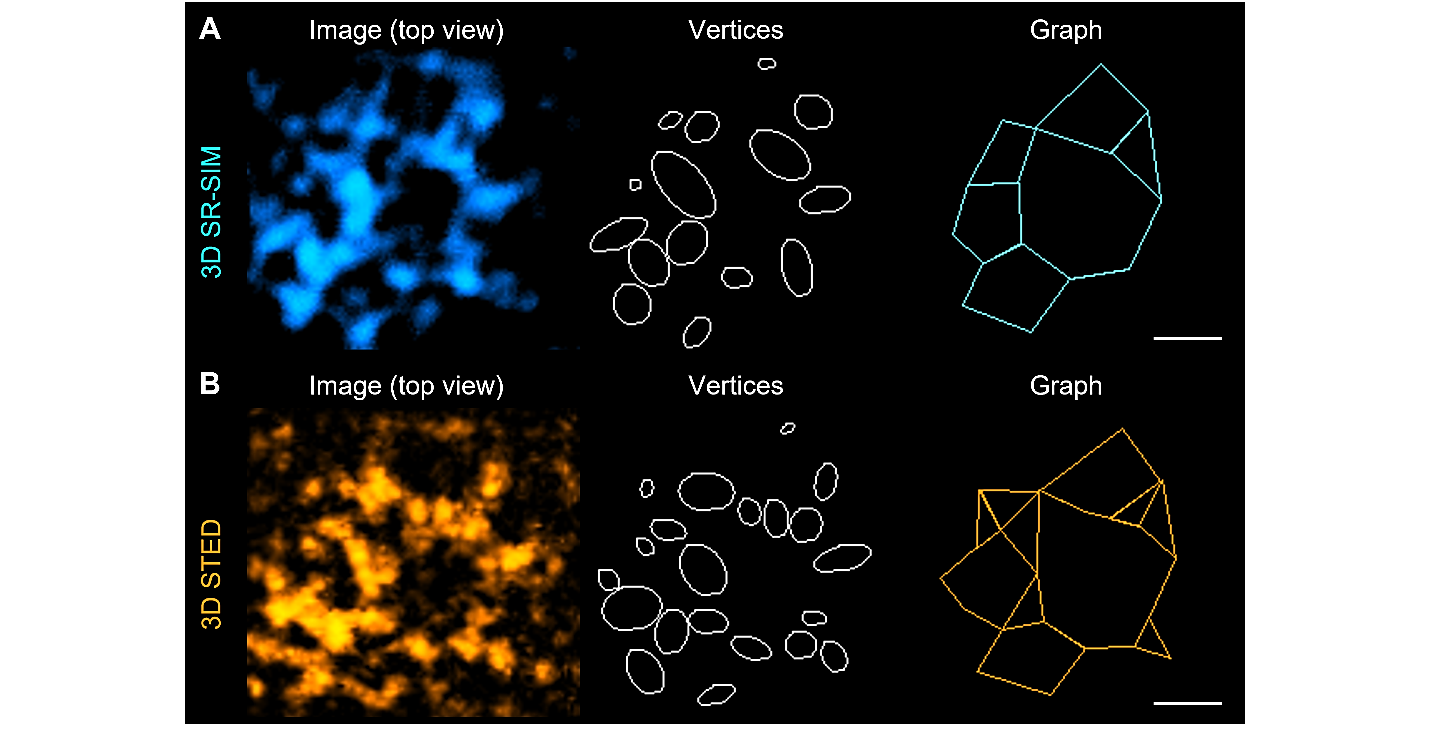


**Figure S4. Structure of a putative synaptic pocket.**

High magnification single plane images, mesh vertices, and the reconstructed graphs are shown for the same region visualized using (**A**) SR-SIM and (**B**) STED microscopy. Scale bars, 500 nm.


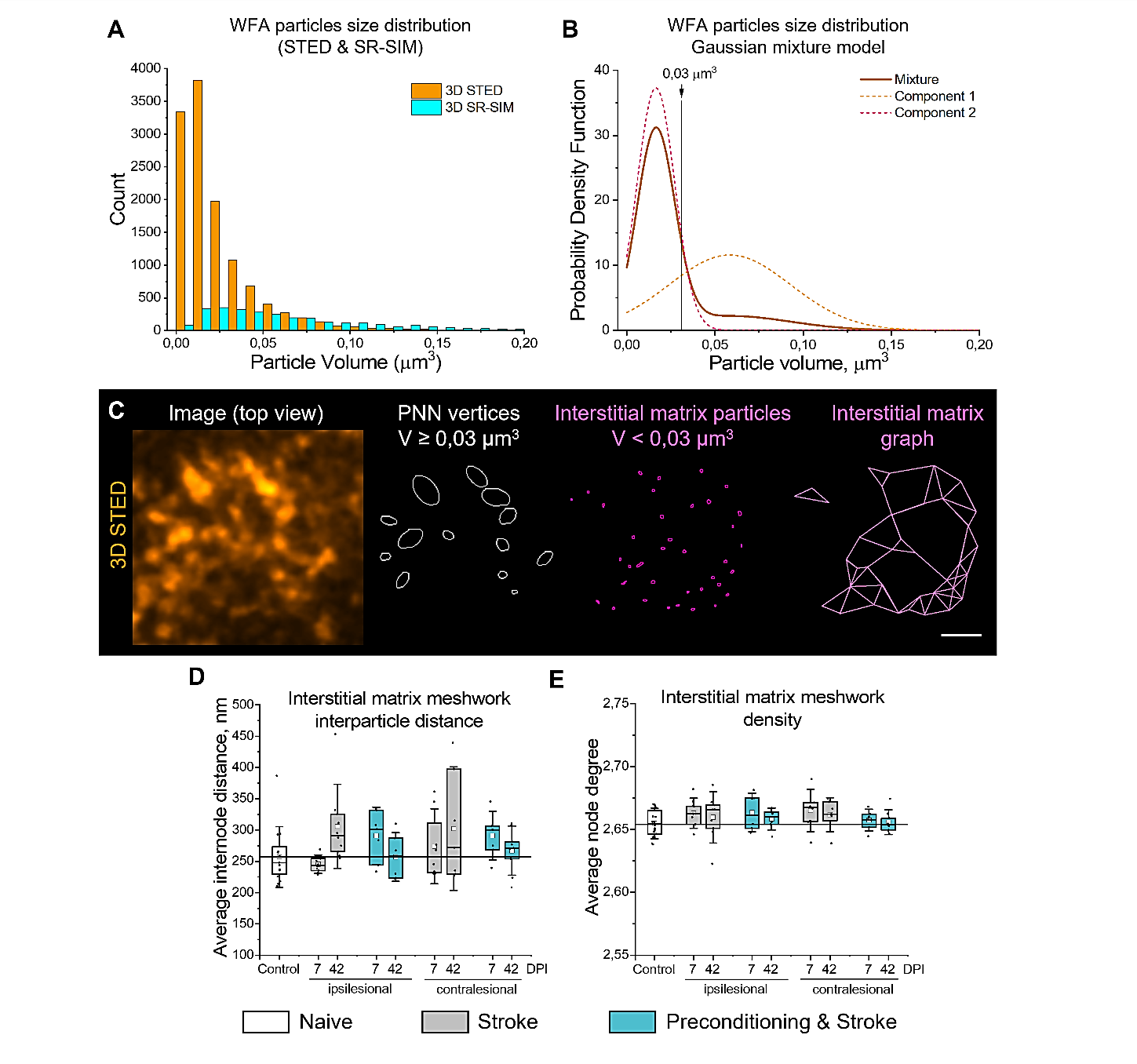


**Figure S5. STED microscopy detects both PNN vertices and interstitial matrix particles.**

(**A**) Histograms show the distribution of WFA-labeled particle volumes measured with SR-SIM and STED microscopy in control mice (n=5). Bin size, 0.01 µm^3^. (**B**) Gaussian mixture model indicates the bimodal distribution of PNN vertex volumes measured by STED microscopy. The 0.03 µm^3^ cutoff was chosen to filter the small vertices not associating with PNNs. (**C**) High magnification single plane STED image, PNN vertices, interstitial matrix particles, and the reconstructed interstitial matrix graph are shown. Scale bars, 500 nm. Interparticle distance (**D**) and meshwork density (**E**) quantifications indicate no significant alterations in the interstitial matrix post stroke. Graphs are box plots with data as dots, means as squares, medians as lines, interquartile ranges as boxes and whiskers showing SD. n=5. DPI, days post ischemia.


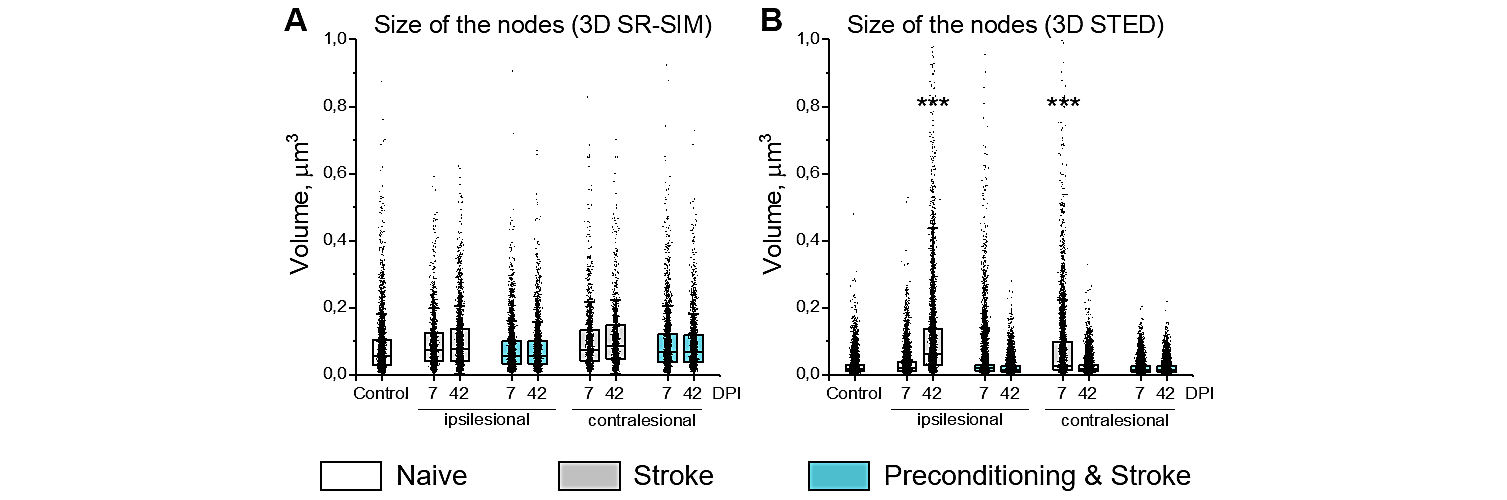


**Figure S6. Size of PNN nodes post stroke.**

The size of PNN nodes was measured with superresolution (**A**) SR-SIM and (**B**) 3D STED microscopy as the volume of ellipsoids. Graphs are box plots with data as dots, means as squares, medians as lines, interquartile ranges as boxes and whiskers showing SD. Asterisks denote significant differences with the control group, as indicated by two-way ANOVA and t-tests (***p < 0.001), n=5. DPI, days post ischemia.


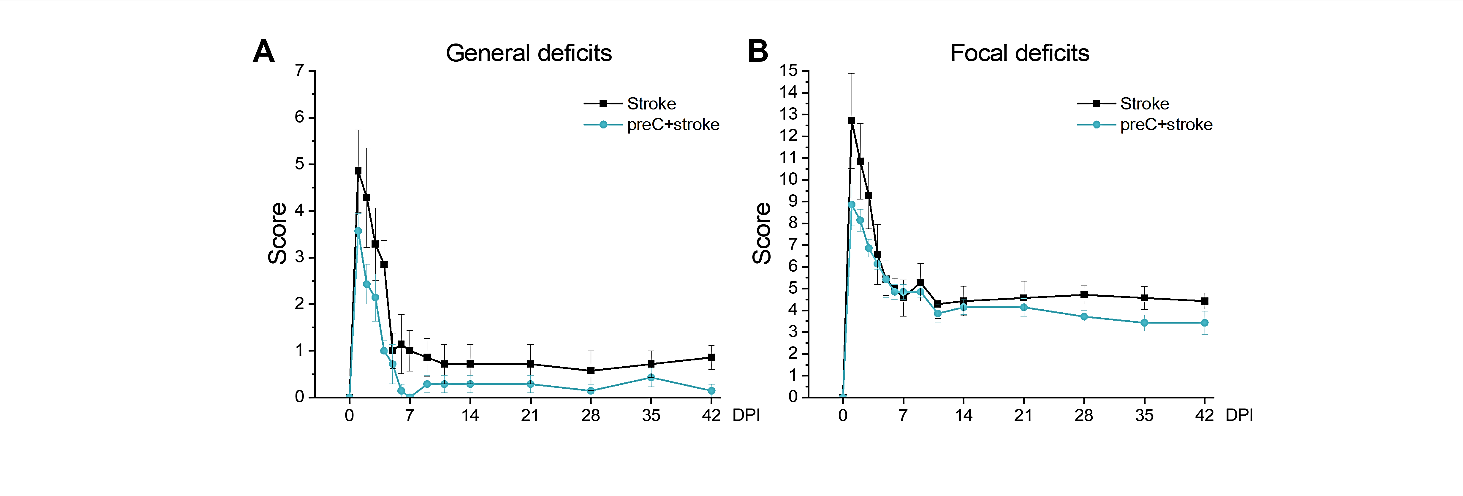


**Figure S7. Neurological deficits post stroke.**

(**A**) Clark’s general deficits scoring. (**B**) Clark’s focal deficits scoring. Data are mean±s.e.m. n = 7. DPI, days post ischemia.
